# Integrative Transcriptomic and Metabolomic Analysis Reveals the Molecular Regulatory Mechanisms of Leaf Rust Resistance in *Zanthoxylum armatum*

**DOI:** 10.3390/biology15181638

**Published:** 2026-09-16

**Authors:** Hanlu Hu, Chengjie Shu, Xiaoxia Sun, Lei Wang, Hao Zhang, Peina Zhou, Xiaode Huang

**Affiliations:** China CO-OP Nanjing Institute for Comprehensive Utilization of Wild Plants, Nanjing 211111, China

**Keywords:** *Zanthoxylum armatum*, leaf rust, transcriptome, widely targeted metabolomics, AP2/ERF transcription factor

## Abstract

Leaf rust severely limits the yield and quality of *Zanthoxylum armatum* (*Z. armatum*). This study compared a rust-susceptible line (S-YF) and a naturally bud-sport-derived resistant line (R-YF) originating from the same mother plant, using physiological assays, widely targeted metabolomics, and transcriptomics to reveal the underlying resistance mechanisms. The total flavonoids and lignin were significantly higher in R-YF. A total of 298 differentially accumulated metabolites (DAMs) were identified, with enrichment of flavonoids and other defense-related secondary metabolites in R-YF. These compounds were primarily associated with the phenylpropanoid and flavonoid biosynthetic pathways. High-performance liquid chromatography (HPLC) confirmed elevated vitexin, rutin, and hydroxy-β-sanshool accumulation in R-YF, while transcriptome profiling revealed 1117 differentially expressed genes (DEGs). R-YF showed activated pathways associated with phenylpropanoid–flavonoid biosynthesis and stress signaling, which upregulated the structural genes involved in flavonoid production and the key *BAHD* acyltransferase gene responsible for hydroxy-β-sanshool synthesis. The AP2/ERF transcription factor family was identified as central to this process, revealing 284 *ZaAP2/ERF* members that demonstrated clear phylogenetic relationships, conserved domains, nuclear localization, tandem duplication expansion, and promoter enrichment for stress and secondary metabolism-related cis-elements. RT-qPCR was used to validate the expression patterns, indicating that *ZaAP2/ERF* positively regulated rust resistance. Overall, R-YF may reconfigure transcriptional and metabolic networks through *ZaAP2/ERF* to enhance the flavonoid and hydroxy-β-sanshool pathways. This resulted in the effective accumulation of defense metabolites, representing a potential key mechanism for resistance. This study provides a basis for the dissection of molecular mechanisms, resistance gene mining, and molecular breeding in *Z. armatum*.

## Simple Summary

*Zanthoxylum armatum* is an economically important tree species in Southwest China valued for the flavor and medicinal properties of its fruits and leaves. Leaf rust disease severely threatens its cultivation. We discovered a spontaneous branch mutant with strong rust resistance on an otherwise susceptible individual. In this study, the resistant mutant (R-YF) and susceptible material (S-YF) were compared via physiological detection and metabolome and transcriptome analyses. The resistant mutant accumulated greater amounts of lignin for cell wall reinforcement, alongside higher levels of antifungal flavonoids. Vitexin, rutin, hyperoside, hydroxy-α-sanshool, hydroxy-β-sanshool, and hydroxy-γ-sanshool were significantly enriched in R-YF leaves. The AP2/ERF transcription factor family was identified as key regulators mediating defense responses. The resistant mutant achieves rust resistance by strengthening physical defense through cell wall reinforcement and by enhancing chemical defense through the accumulation of antimicrobial metabolites, under the control of AP2/ERF members. This research elucidates the molecular basis of natural rust resistance and supplies candidate targets for *Zanthoxylum armatum* disease-resistance breeding.

## 1. Introduction

*Zanthoxylum armatum* DC. (*Z. armatum*) is an economically important tree species belonging to the *Zanthoxylum* genus in the Rutaceae family. This tree, native to southwestern China, is valued for its fruits and leaves, which serve both culinary and medicinal purposes [1]. A significant product extracted from its fruits is green Sichuan pepper oil, which exhibits a characteristic numbing sensation that is primarily conferred by amides such as hydroxy-α-sanshool and hydroxy-β-sanshool [2]. While previous studies have characterized its culinary uses, aroma components, bioactive substances, and pharmacological activities [3,4,5,6,7,8], the present study focuses on its disease-resistance mechanisms. However, the prevalence of diseases and pests has become increasingly severe due to the rapid expansion of *Z. armatum* production. One of the most significant threats is leaf rust, caused by *Coleosporium zanthoxyli* Dietel & P. Syd., which has become prevalent in key cultivation areas. Infection causes extensive leaf chlorosis and necrosis, while drastically reducing photosynthetic capacity. This adversely affects the yield and quality of pepper fruits, posing a serious challenge to the sustainable development of the *Z. armatum* industry [9,10]. While current chemical treatments for controlling rust disease act quickly, long-term pesticide use tends to cause pathogen resistance and residue pollution, while cultural and biological management practices present various inherent limitations [11]. Screening resistant germplasm and dissecting endogenous plant defense mechanisms represent the optimal strategies for achieving eco-friendly, source-directed disease control. To date, the molecular mechanisms underlying rust resistance in *Z. armatum* remain poorly understood. This study aims to fill this gap and provide a theoretical basis for breeding disease-resistant *Z. armatum* germplasm.

Plants rely on physical barriers and secondary metabolites as defense mechanisms against fungal threats [12,13]. The cell wall serves as a key interface in plant–fungus interactions, acting as both a physical and chemical barrier against pathogen invasion [14]. Lignin deposition thickens the cell wall and blocks pathogen hyphal penetration [15]. Plants naturally synthesize secondary metabolites with direct antimicrobial activity, such as flavonoids and sanshools, for chemical defense. Flavonoids enhance plant disease resistance through both direct antimicrobial activity and the activation of plant defense pathways [16]. Lignin and flavonoids are both derived from phenylalanine via the phenylpropanoid pathway. Key enzymes, including phenylalanine ammonia-lyase (*PAL*), cinnamate 4-hydroxylase (*C4H*), and 4-coumarate-CoA ligase (*4CL*), convert phenylalanine into coumaroyl-CoA, a common branch-point precursor. This precursor then diverges into two pathways: one for lignin biosynthesis, producing lignin polymers, and the other for catalysis by flavonoid skeleton enzymes such as chalcone synthase (*CHS*), chalcone isomerase (*CHI*), and flavone synthase (*FNS*), ultimately generating various flavonoid derivatives, including vitexin and rutin [17,18,19]. Unlike lignin and flavonoids, hydroxy-β-sanshool is a characteristic unsaturated fatty acid amide. Its biosynthesis relies on branched-chain amino acid degradation and unsaturated fatty acid pathways to supply amine precursors and the acyl skeleton, respectively, followed by condensation catalyzed by *BAHD* acyltransferases [20]. Although progress has been made in elucidating the biosynthetic flavonoid and amide pathways in *Z. armatum*, the regulation of these processes by transcription factors and their roles in the response to biotic stress remain unclear.

AP2/ERF proteins represent a significant superfamily of transcription factors unique to plants, playing essential roles in various physiological processes, including growth and development, hormone signal transduction, stress responses, and secondary metabolism regulation [21,22,23]. The family is classified into five major subfamilies based on domain architecture and sequence divergence: DREB, ERF, AP2, RAV, and Soloist [24]. The AP2 subfamily contains two tandem AP2 domains and mainly regulates organ morphogenesis and seed development [25,26]; the RAV subfamily possesses both AP2 and B3 domains and responds to hormone signals and stress [27,28,29]; the Soloist subfamily has low sequence conservation and participates in defense via phenylpropanoid metabolism [30]; the DREB subfamily mainly mediates abiotic stress responses, with prominent functions in cold-stress adaptation [31]; and the ERF subfamily, the largest, responds to ethylene, jasmonic acid and salicylic acid and broadly regulates biotic and abiotic stress responses [32,33,34,35,36,37]. Regarding their regulatory mechanisms, DREB members mainly recognize DRE/CRT cis-elements to activate downstream abiotic stress-responsive gene expression [38,39]. ERF members primarily bind the GCC-box (GCCGCC) and other ethylene-responsive elements to regulate stress-related gene transcription, functioning as either transcriptional activators or repressors [40,41]. Previous studies have demonstrated that ERF members bind to GCC-box motifs in the promoters of key flavonoid biosynthesis genes, such as *4CL*, *CHS*, and *CHI*, to regulate flavonoid accumulation. Moreover, the AP2/ERF family coordinately regulates stress responses and secondary metabolism in plants [42,43]. Two classification systems divide DREB and ERF into subclades with distinct gene structures and conserved motifs associated with functional divergence [44,45].

Most existing studies on *Z. armatum* disease resistance have focused on individual metabolites or single genes. Minimal integrated multi-omics analyses are available that combine transcriptomics and metabolomics to decipher the overall regulatory rust-resistance network in leaves. Furthermore, a genome-wide systematic characterization of the core regulatory transcription factor families has yet to be performed. We hypothesized that rust-induced *ZaAP2/ERF* transcription factors coordinate the activation of flavonoid and hydroxy-β-sanshool biosynthesis, leading to enhanced antimicrobial metabolite accumulation and increased physical and chemical defense, ultimately conferring leaf rust resistance in *Z. armatum*. This study identified a susceptible line (S-YF) and a resistant bud sport mutant (R-YF) originating from the same mother plant. The use of these paired materials helps reduce genetic background interference, although the extent of somatic variation between the two lines remains to be further characterized. The physiological and biochemical indices were measured, followed by widely targeted metabolomics and transcriptome sequencing to analyze the content changes in key bioactive compounds (sanshools and flavonoids) and the gene expression related to their biosynthesis in S-YF and R-YF. Gene family analysis and expression profiling identified *ZaAP2/ERF* members that might regulate secondary metabolite biosynthesis and disease resistance. This study offers essential insights into the molecular mechanisms underlying rust resistance and lays the groundwork for identifying potential resistance genes in *Z. armatum*.

## 2. Materials and Methods

### 2.1. Plant Materials

This study used two distinct lines: S-YF, a rust-susceptible line, and R-YF, a naturally resistant bud sport mutant derived from the same parent plant in the same cultivation plot. During the natural peak of rust disease in the field, three spatially separated resistant bud-sport branches of R-YF and three independent susceptible branches of S-YF were sampled, with each branch serving as one biological replicate. Leaves collected from each branch were pooled to form one biological replicate. The leaves were immediately frozen in liquid nitrogen and stored at −80 °C for further use. Because the two lines originated from the same mother plant, this paired material was expected to reduce genetic background variation; however, the extent of somatic variation between S-YF and R-YF remains to be characterized at the genomic level. Rust resistance was evaluated based on qualitative field symptoms; quantitative disease indices, spore germination assays, and controlled inoculation experiments were not performed in the present study.

### 2.2. Reagents and Instruments

Chromatographic-grade standards (HPLC ≥ 98%) of vitexin, rutin, hyperoside, isoquercitrin, hydroxy-α-sanshool, hydroxy-β-sanshool, and hydroxy-γ-sanshool were purchased from Sichuan Weikeqi Biotechnology Co., Ltd. (Chengdu, China). The total flavonoid and lignin detection kits were obtained from Beijing Solarbio Science & Technology Co., Ltd. (Beijing, China), while Nanjing Vazyme Biotech Co., Ltd. (Nanjing, China) supplied the plant polysaccharide–polyphenol RNA extraction, reverse transcription, and SYBR Green real-time PCR kits. The HPLC-grade methanol and acetonitrile were purchased from Merck (Darmstadt, Germany), and the chromatographic-grade formic acid was provided by Sigma-Aldrich (St. Louis, MO, USA). All analytical equipment and reagents for multi-omics were as follows. For HPLC metabolite detection, a Cary 500 UV-Vis spectrophotometer (Agilent, Santa Clara, CA, USA) and an Agilent LC 1260 Infinity II (Agilent Technologies, Santa Clara, CA, USA) HPLC system equipped with a VWD detector and a C18 column were used. The transcriptome platform included a Qubit 4.0 fluorometer (Thermo Fisher Scientific, Waltham, MA, USA), a Qsep400 capillary analyzer (BiOptic, New Taipei, Taiwan, China), a DNBSEQ sequencer (MGI, Shenzhen, China), and a CFX96 Touch real-time PCR system (Bio-Rad, Hercules, CA, USA). Widely targeted metabolomics was performed on an ExionLC™ AD UHPLC coupled with a QTRAP 450 MS/MS (AB Sciex, Concord, ON, Canada). Auxiliary equipment included an MM 400 grinder (Retsch, Haan, Germany), a Scientz-100F lyophilizer (Ningbo Scientz Biotechnology, Ningbo, China), and a 5424R centrifuge (Eppendorf, Hamburg, Germany). HPLC-grade methanol, acetonitrile, and formic acid were purchased from Merck (Darmstadt, Germany). Transcriptome data were processed using Fastp (version 0.23.2) and HISAT2 (version 2.2.1), and metabolomic mass spectra were integrated with Analyst 1.6.3. The analytical software and tools used in this study included TBtools (version 2.311), MEGA 6, iTOL (https://itol.embl.de, accessed on 25 June 2026), Origin (version 2024b), MultiQuant (version 3.0.3), GraphPad Prism (version 9.0), and the MEME Suite (version 5.5.9).

### 2.3. Experimental Methods

#### 2.3.1. Determination of the Total Flavonoid and Lignin Content

Total flavonoids in leaf samples were extracted by ultrasonic-assisted treatment using 70% ethanol solution. Quantification was conducted via the sodium nitrite–aluminum nitrate colorimetric assay, where rutin served as the reference standard [46]. Standard calibration curves were generated based on absorbance readings recorded at 510 nm after chromogenic reactions. The lignin content was measured using UV spectrophotometry according to the Solarbio kit instructions. All measurements were performed with *n* = 3 biological replicates per genotype.

#### 2.3.2. HPLC Quantification of Flavonoids and Hydroxy Sanshools

Ground leaf samples (1 g, accurately weighed to 0.0001 g) were extracted with 10 mL of methanol by vortexing for 1 min and ultrasonication for 30 min, then centrifuged at 4000 rpm for 10 min and filtered through a 0.22 μm membrane before HPLC injection. For vitexin, separation was performed on an Origsil C18 column (Beijing Naou Technology Co., Ltd., Beijing, China) (5 μm, 4.6 × 150 mm) with 0.1% formic acid in water (A) and acetonitrile (B) at 85:15 (*v*/*v*), at 1.0 mL/min, with detection at 330 nm over 22 min. Hyperoside was analyzed after 10-fold dilution of the supernatant using the same column type with 0.1% phosphoric acid in water (A) and acetonitrile (B) at 70:30 (*v*/*v*), at 1.0 mL/min, with detection at 360 nm over 10 min. Rutin and isoquercitrin were determined with 0.1% phosphoric acid in water (A) and acetonitrile (B) at 80:20 (*v*/*v*), at 1.0 mL/min, with detection at 360 nm over 15 min. Hydroxy-β-sanshool was measured on a Welch Ultimate AQ-C18 column (Welch Materials, Inc., Shanghai, China) (5 μm, 4.6 × 250 mm) with pure water (A) and acetonitrile (B) at 35:65 (*v*/*v*), at 1.0 mL/min, with detection at 270 nm over 10 min. Hydroxy-α-sanshool and hydroxy-γ-sanshool were measured on an Agilent ZORBAX SB-Aq column (Agilent Technologies, Santa Clara, CA, USA) (3.5 μm, 2.1 × 150 mm) with pure water (A) and acetonitrile (B) at 60:40 (*v*/*v*), at 0.40 mL/min, with detection at 270 nm over 15 min.

#### 2.3.3. Widely Targeted Metabolomics

The freeze-dried leaves were ground, followed by extraction with 70% methanol at 4 °C. After centrifugation at 12,000× *g* for 10 min at 4 °C and filtration, the extracts were separated on an Agilent SB-C18 column (Agilent Technologies, Santa Clara, CA, USA) using gradient elution and analyzed on an AB 4500 QTRAP mass spectrometer (AB Sciex, Concord, ON, Canada) in ESI positive and negative ion modes. The MWDB database was used for metabolite identification, while MultiQuant was employed for quantification. The differential metabolites were screened using criteria of VIP > 1 and log_2_fold-change (log_2_FC) ≥ 2, followed by classification and KEGG enrichment analysis [47].

#### 2.3.4. Transcriptome Sequencing and Bioinformatics Analysis

Total RNA was isolated from leaf samples of *Z. armatum* using the plant polysaccharide–polyphenol-rich total RNA extraction kit (Vazyme Biotech, Nanjing, China). RNA concentration was quantified with a Qubit 4.0 fluorometer, and RNA integrity was assessed using a Qsep400 biofragment analyzer. RNA samples meeting the quality criteria (RQN ≥ 6.5, OD260/280 ranging from 1.8 to 2.2) were retained for cDNA library construction. Qualified libraries were sequenced on the DNBSEQ-T7 platform (MGI-Tech, Shenzhen, China). Raw sequencing reads were filtered by fastp software: adapter-containing reads were removed; paired-end reads were discarded if any read contained more than 10% ambiguous N bases; read pairs were also filtered out when over 50% of bases within one read had Phred quality ≤ 20. The resulting clean reads were mapped to the *Z. armatum* reference genome using HISAT2. Gene-level read counts were calculated with featureCounts, and gene expression values were estimated as FPKM. DEGs were screened using DESeq2 (version 1.22.1). Genes with log_2_FC ≥ 1 and adjusted *p*-value (FDR) < 0.05 were defined as up-regulated, while genes with log_2_FC ≤ −1 and FDR < 0.05 were defined as down-regulated. The terms “up-regulated” and “down-regulated” are strictly reserved for genes satisfying the above genome-wide DEG thresholds. Subsequent pathway-level analyses included all annotated biosynthetic-pathway-related genes for comprehensive inspection, including genes that do not satisfy the DEG criteria; for these genes, we use the neutral descriptions “higher expression” or “lower expression” instead of DEG-related terminology. Given that R-YF and S-YF originate from the same mother tree, modest transcript-level shifts of pathway-associated genes may collectively contribute to metabolic phenotypic changes. GO enrichment, KEGG enrichment, and transcription-factor classification analyses were further performed on the DEG dataset [48]. Three biological replicates were used for each genotype.

#### 2.3.5. Genome-Wide Bioinformatics Analysis of the AP2/ERF Transcription Factor Family in *Z. armatum*

The genome sequence and the corresponding gff3 annotation file of *Z. armatum* were obtained from the Figshare platform. Using the *Arabidopsis thaliana* AP2/ERF protein sequences as queries, local BLAST (https://ftp.ncbi.nlm.nih.gov/blast/executables/blast+/LATEST/, accessed on 8 September 2026) searches were performed with TBtools at a threshold of E-value < 1 × 10^−3^. The AP2 conserved domain model PF00847 was downloaded from the Pfam database, and a hidden Markov model (HMM) search was conducted using the Simple HMM Search tool in TBtools with a threshold of E-value < 1 × 10^−10^. The results from both approaches were combined, and redundant sequences were removed, yielding 284 candidate AP2/ERF protein sequences [49]. The Protein Parameter Calc module in TBtools was used to batch-calculate the physicochemical properties, including the amino acid lengths, molecular weights, theoretical isoelectric points, aliphatic indices, and grand averages of hydropathicity of all family proteins [50]. The Show Gene on Chromosome tool was employed to visualize the chromosomal distribution of the family genes, while the conserved motifs and characteristic domains were integrated and displayed using the Gene Structure View (Advanced) module [51]. The full-length *Z. armatum* and A. thaliana AP2/ERF protein sequences were imported into TBtools to construct a maximum likelihood (ML) phylogenetic tree. After clustering, the tree was refined and visualized using the iTOL website.

#### 2.3.6. RT-qPCR Validation

Based on transcriptome screening, the key genes in the flavonoid biosynthetic pathway were selected, including shikimate O-hydroxycinnamoyltransferase (*HCT*), *4CL*, scopolin glucosyltransferase (*TOGT1*), cinnamyl alcohol dehydrogenase (*CAD*), flavanone 3-hydroxylase (*F3H*), and anthocyanidin reductase (*ANR*). The representative *BAHD* acyltransferase family genes in the hydroxy-β-sanshool biosynthetic pathway were also selected, along with the *ZaAP2/ERF* transcription factor genes. All primer sequences are listed in Table S1. Total RNA was extracted from the leaves and reverse-transcribed into cDNA. β-Actin was selected as the internal reference gene because its raw Ct values showed small variation between S-YF and R-YF samples, and it has been commonly used as a reference gene in Zanthoxylum expression studies [52,53]. The relative gene expression was calculated using the 2^−ΔΔCt^ method, and differences in expression between the two groups were assessed using an independent-samples *t*-test [54]. All measurements were performed with *n* = 3 biological replicates per genotype.

#### 2.3.7. Data Analysis

Quantitative phenotypic and RT-qPCR data were visualized using GraphPad Prism and Origin. Before statistical testing, normality was assessed using the Shapiro–Wilk test, and homogeneity of variances was evaluated using the Brown–Forsythe test. When these assumptions were satisfied, differences between two groups were evaluated by independent-samples *t*-test, whereas comparisons among multiple groups were performed using one-way ANOVA. When assumptions were violated, Welch’s *t*-test or the Mann–Whitney U test was used for two-group comparisons, while Welch-corrected one-way ANOVA or Kruskal–Wallis test was adopted for multi-group comparisons. For transcriptome analysis, the Benjamini–Hochberg FDR procedure was implemented to correct for multiple comparisons; genes with |log_2_FC| ≥1 and FDR <0.05 were regarded as DEGs. For widely targeted metabolomics, the Benjamini–Hochberg FDR correction was applied to metabolite-level *p*-values derived from significance testing. DAMs were defined by the combined criteria from the OPLS-DA model and statistical testing: VIP > 1, log_2_FC ≥ 2, and FDR < 0.05. For RT-qPCR, a small set of pre-selected candidate genes were analysed by independent-samples *t*-test; given the limited number of targets, additional FDR-based multiple-comparison correction was not performed. Statistical significance levels were denoted as * *p* < 0.05, ** *p* < 0.01, and *** *p* < 0.001 in figures and figure captions.

## 3. Results

### 3.1. Analysis of Total Flavonoid and Lignin Contents in Z. armatum Leaves

**Figure 1:**
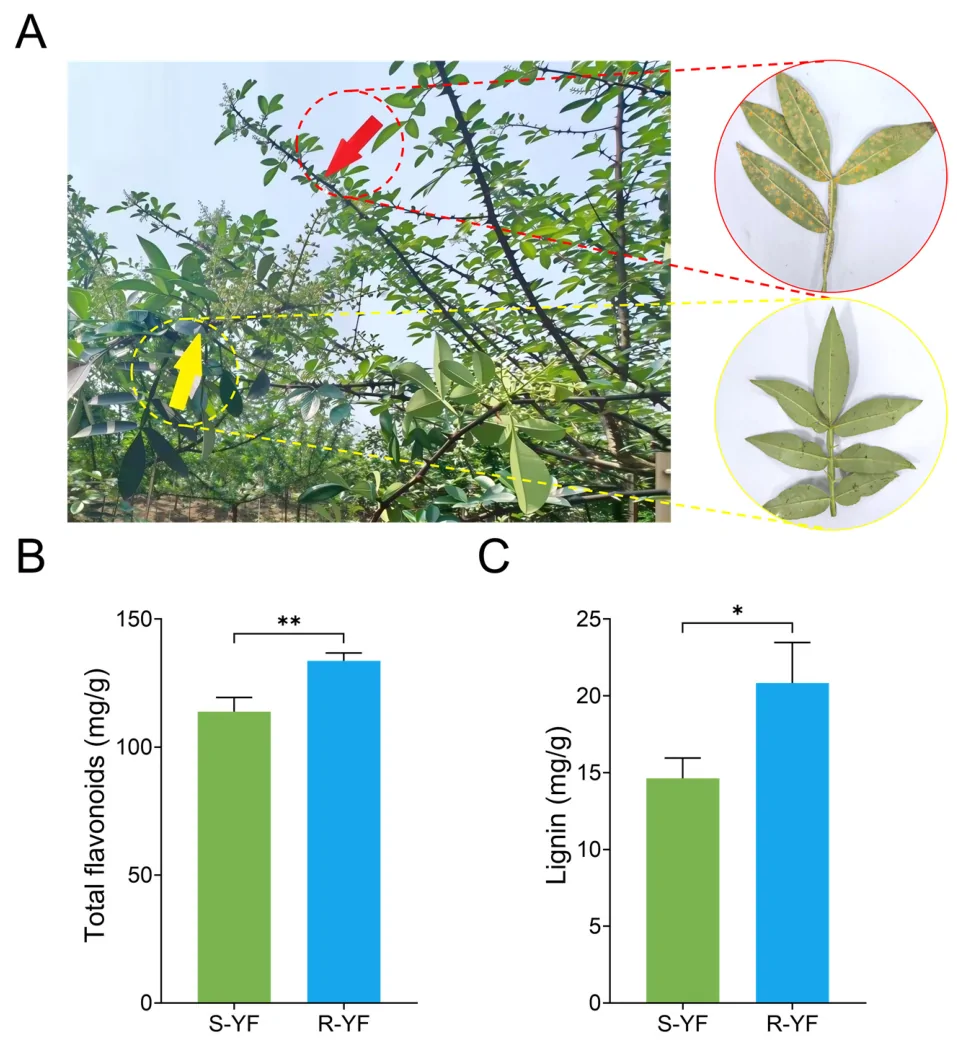
A comparison between the leaf phenotypes, total flavonoid content, and lignin content in S-YF and R-YF. (**A**) Natural rust disease phenotypes in the field. The red circles indicate the susceptible leaves of S-YF, while the yellow circles denote the resistant leaves of R-YF. (**B**) The total flavonoid content in the leaves. (**C**) The lignin content in the leaves. Data are presented as means ± SD (*n* = 3 biological replicates per group). * *p* < 0.05, ** *p* < 0.01.

Field phenotypic observation during the natural peak of rust disease showed that the leaves of S-YF were covered with large areas of orange uredinia and severe chlorotic lesions, while those of R-YF exhibited no obvious rust infection symptoms, demonstrating a clear difference in rust resistance phenotypes (Figure 1A). Lignin is a key cell-wall component that protects against pathogens such as fungi and bacteria, while total flavonoids enhance biotic stress resistance. Therefore, the total flavonoid and lignin content in S-YF and R-YF was determined, showing that the levels of both substances were markedly elevated within R-YF relative to S-YF leaves (Figure 1B,C; *p* < 0.01, *p* < 0.05). These results indicated that secondary antimicrobial flavonoid metabolite accumulation and cell-wall lignin deposition were simultaneously enhanced in R-YF. This established a coordinated basal defense system that combines chemical and physical defenses, denoting its superior field resistance to leaf rust.

### 3.2. Widely Targeted Metabolomic Analysis of Leaves from S-YF and R-YF

**Figure 2:**
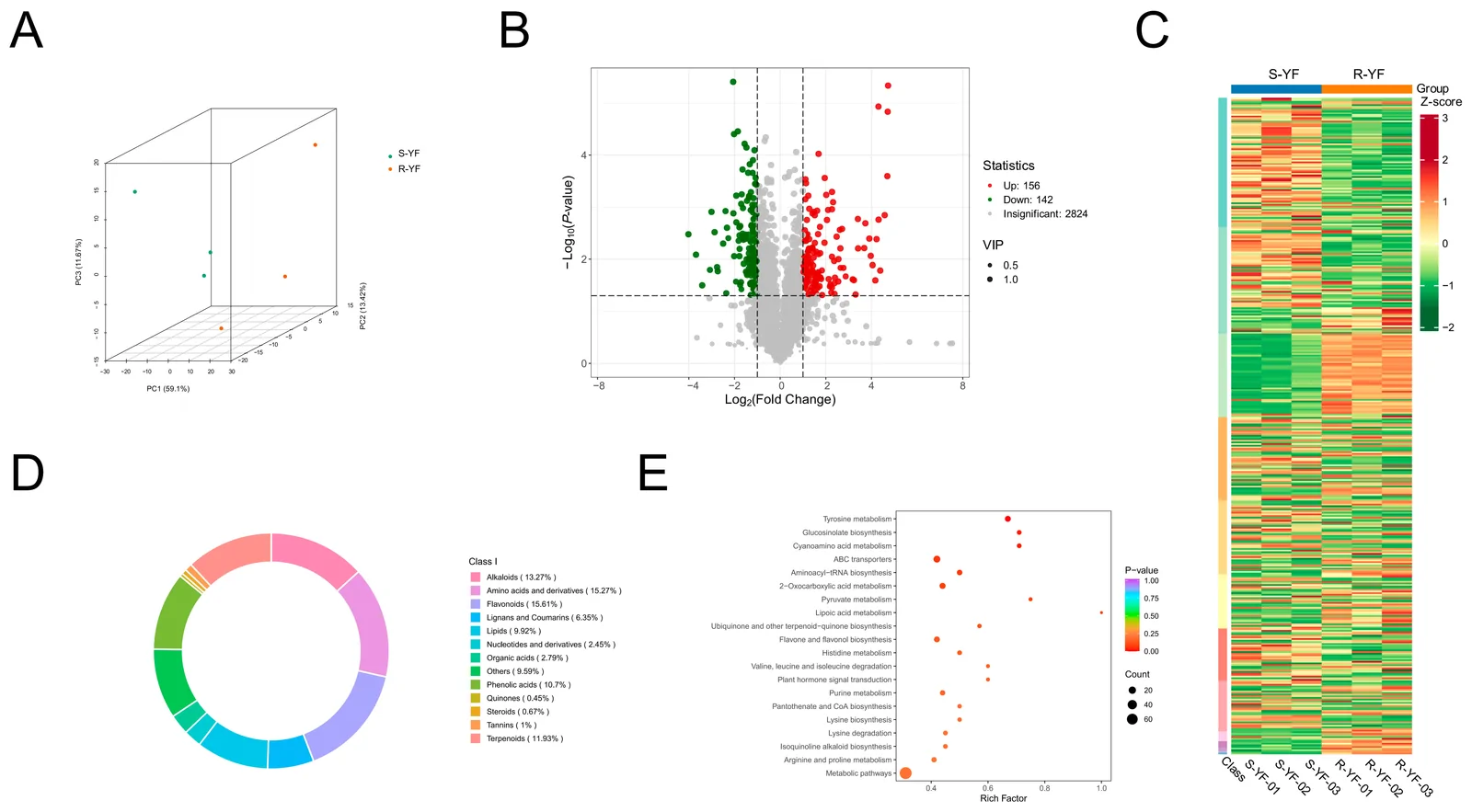
The global, widely targeted metabolomic analysis of the S-YF and R-YF leaves. (**A**) The three-dimensional PCA score plot. (**B**) Volcano visualization for DAM datasets. The dashed horizontal line indicates the significance threshold at −log_10_(0.05) (corresponding to *p* = 0.05), and the dashed vertical lines indicate the log_2_ fold-change thresholds used for screening DAMs (see Section 2.3.2). Red dots stand for metabolites with strong up-regulation, green dots refer to those that are markedly down-regulated, and dot size corresponds to VIP values. (**C**) Hierarchical clustering heatmap of the DAMs. (**D**) Donut chart of the DAM category distribution. (**E**) KEGG enrichment bubble plot. Bubble diameter reflects the DAM count of each pathway, and color intensity indicates enrichment significance.

Widely targeted metabolomic profiling was performed to clarify the distinct metabolic differences between the S-YF and R-YF leaves. Principal component analysis (PCA) (Figure 2A) revealed clear separation of the two sample groups across principal-component axes. The biological replicates in each group were tightly clustered, with no overlap between groups, indicating a considerable difference between the overall metabolic profiles of the S-YF and R-YF leaves, as well as good experimental reproducibility. The volcano plot of the DAMs (Figure 2B) identified 298 DAMs, of which 156 were significantly upregulated, and 142 were significantly downregulated. Most of the DAMs were distributed in regions marked by high fold changes and significance. The hierarchical clustering heatmap (Figure 2C) visually represented variations in DAM patterns between the two groups, with a notable number of metabolites showing markedly higher accumulation in R-YF. The donut chart of the DAM categories (Figure 2D) showed that flavonoids accounted for 15.61% of the total DAMs, followed by amino acids and their derivatives at 15.27%, ranking as the primary categories. Relatively high proportions of amino acid derivatives, terpenoids, phenolic acids, lignans, and coumarins were also evident. The KEGG enrichment bubble plot (Figure 2E) demonstrated abundant differential metabolites within the flavonoid and flavonol biosynthesis, phenylpropanoid biosynthesis, amino acid metabolism, and lipid metabolism pathways. The KEGG functional annotation classification of DAMs is presented in Figure S2. The valine, leucine, and isoleucine degradation pathways were also significantly enriched, providing key precursors for hydroxy-β-sanshool synthesis. These results imply that R-YF reconstructs leaf secondary-metabolite networks and triggers the buildup of defensive compounds including flavonoids, thereby establishing a chemical defense signature against leaf rust infection.

### 3.3. HPLC Quantification of the Characteristic Flavonoids and Hydroxy-Sanshool Monomers

**Figure 3:**
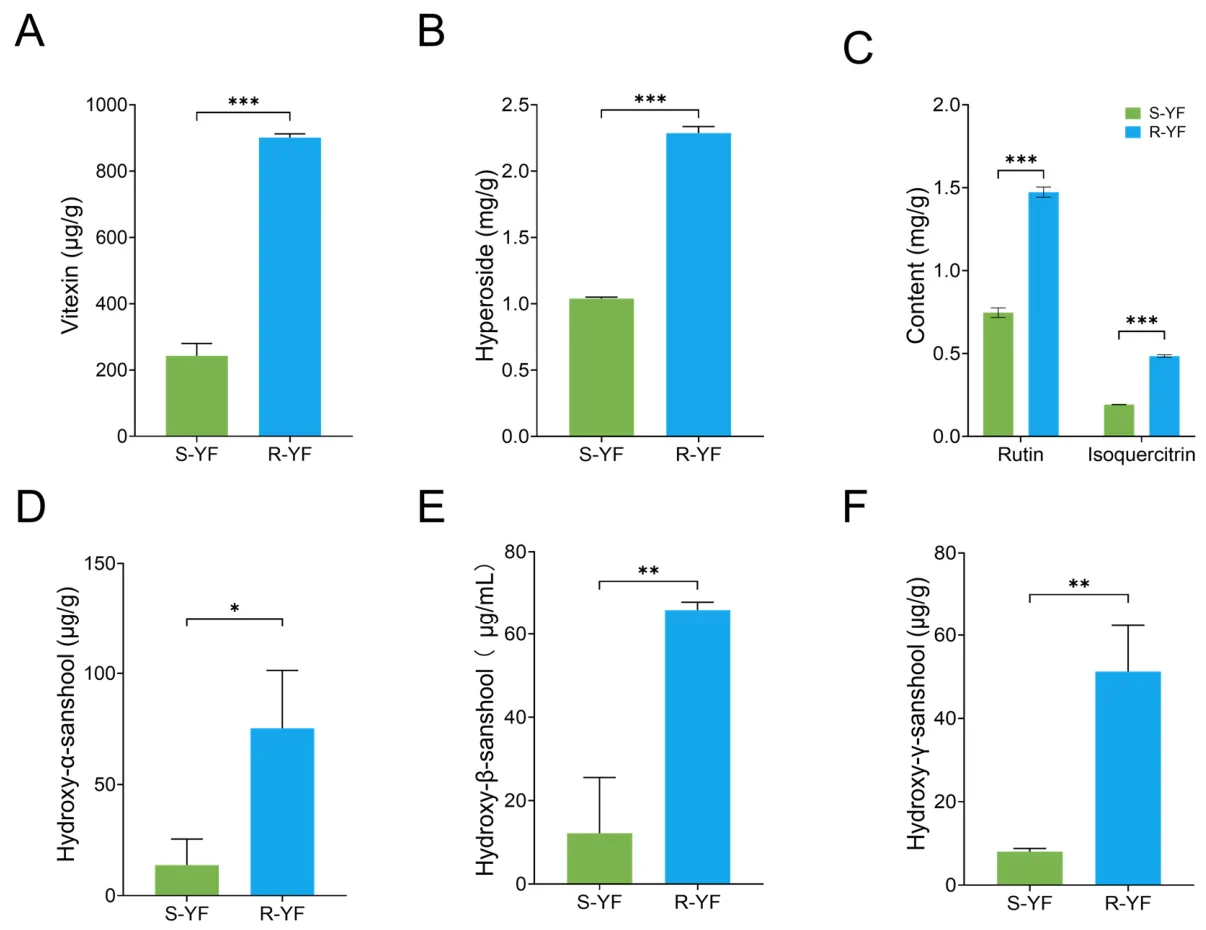
The HPLC quantification of the characteristic flavonoids and hydroxy sanshools in the S-YF and R-YF leaves. Green bars represent S-YF (rust-susceptible line), and blue bars represent R-YF (rust-resistant bud-sport line). (**A**) Vitexin content. (**B**) Hyperoside content. (**C**) Rutin and isoquercitrin content. (**D**) Hydroxy-α-sanshool content. (**E**) Hydroxy-β-sanshool content. (**F**) Hydroxy-γ-sanshool content. Data are presented as means ± SD (*n* = 3 biological replicates per group). * *p* < 0.05, ** *p* < 0.01, *** *p* < 0.001.

An HPLC external standard method was employed to quantify the levels of four characteristic flavonoid monomers and three hydroxy sanshools in the leaves; the representative chromatograms of standards and leaf samples are shown in Figure S1. As shown in Figure 3, the vitexin, hyperoside, rutin, isoquercitrin, hydroxy-α-sanshool, hydroxy-β-sanshool, and hydroxy-γ-sanshool accumulation levels were significantly higher in the R-YF leaves than in those of S-YF (*p* < 0.05, *p* < 0.01, and *p* < 0.001). The HPLC results were consistent with the metabolomic enrichment analysis and total flavonoid measurements, indicating that R-YF could accumulate antimicrobial flavonoid polyphenols and characteristic amide metabolites such as hydroxy sanshools. Together, these two classes of bioactive compounds played a crucial role in establishing a chemical defense against leaf rust infection in *Z. armatum*.

### 3.4. Transcriptome Analysis

**Figure 4:**
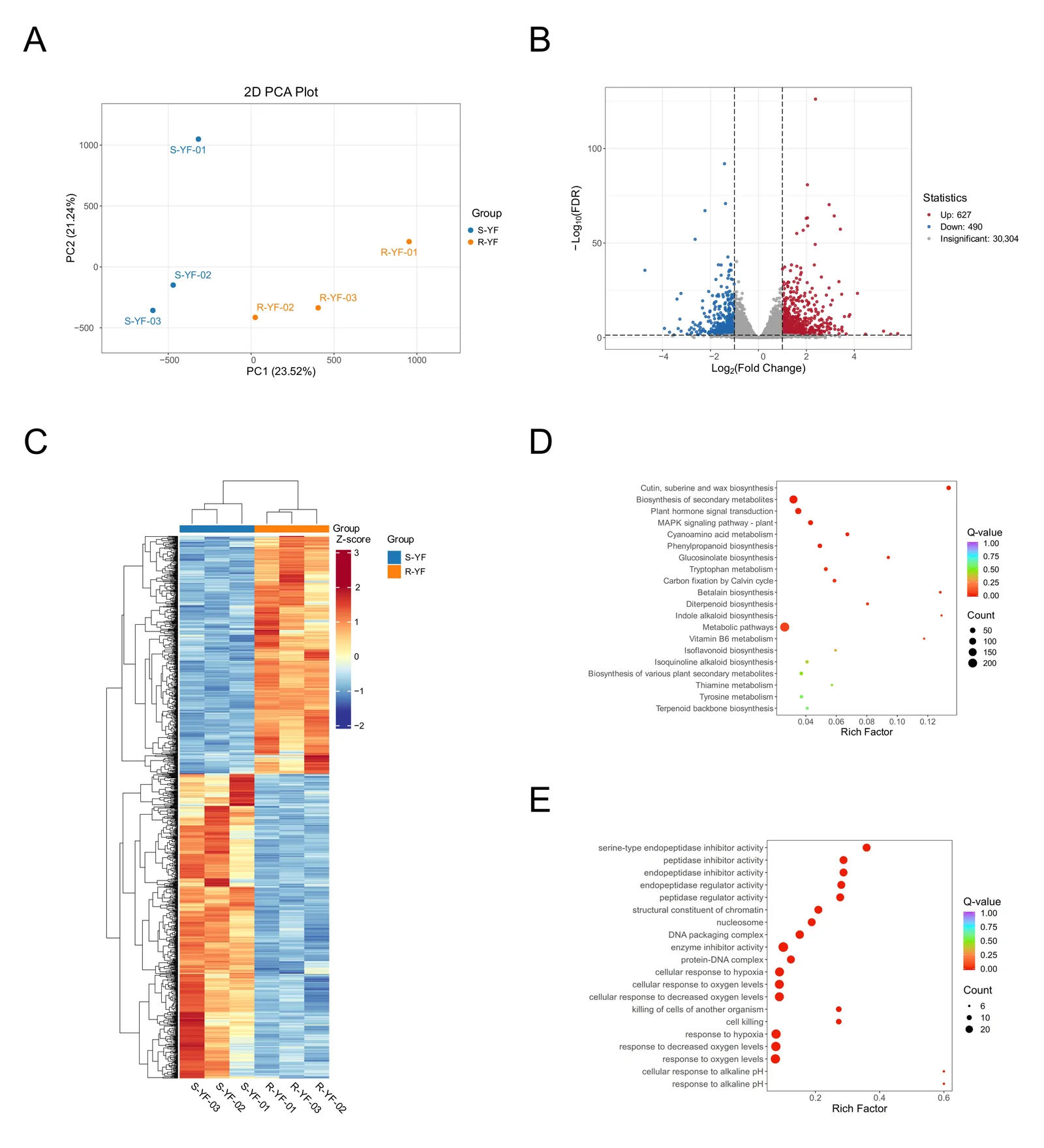
The global transcriptome analysis of the S-YF and R-YF leaves. (**A**) Two-dimensional PCA score plot. (**B**) Volcano plot of the DEGs. The dashed horizontal line indicates the significance threshold at −log_10_(0.05) (corresponding to *p* = 0.05), and the dashed vertical lines indicate the log_2_ fold-change thresholds (log_2_FC = −1 and 1). (**C**) Hierarchical clustering heatmap of the DEGs. (**D**) KEGG enrichment bubble plot of the DEGs. (**E**) GO enrichment bubble plot of the DEGs.

The S-YF and R-YF leaf samples underwent transcriptome sequencing to unravel the transcriptional differences between these groups. Two-dimensional PCA (Figure 4A) confirmed good reproducibility of the sequencing samples in each group. According to the volcano plot (Figure 4B), 1117 significant DEGs were screened out across the two groups, with 627 genes up-regulated, whereas 490 genes displayed reduced expression. The hierarchical clustering heatmap of the DEGs (Figure 4C) visually represented the distinct gene expression patterns between the two lines. A significant number of genes linked to secondary metabolite biosynthesis and biotic-stress-adaptive responses were highly expressed in R-YF. The KEGG enrichment bubble plot (Figure 4D) showed that defense-related pathways, such as cutin, suberine and wax biosynthesis, secondary metabolite biosynthesis, and plant hormone signal transduction ranked high in both enrichment significance and factor. Notably, the DEGs exhibited prominent enrichment within secondary metabolic routes, including phenylpropanoid, flavonoid, and flavonol biosynthesis. The KEGG annotation bar chart (Figure S3) indicated that secondary-metabolite formation, plant–pathogen crosstalk, and phytohormone signal transduction contributed the greatest fraction of DEGs. This suggests that rust stress primarily modulates gene transcription related to secondary metabolism and defense signaling. The GO enrichment bubble plot (Figure 4E) showed that stress defense-related terms, such as serine-type endopeptidase inhibitor activity, chromatin assembly, cellular response to hypoxia, and cell killing, were significantly enriched. The GO classification histogram (Figure S4) illustrated that the DEGs assignments fell mainly into three major categories: biological processes (BP), cellular components (CC), and molecular functions. In these categories, the most annotated terms were cellular processes, protein-containing complexes, and binding, respectively. These results indicated that R-YF remodeled the overall transcriptional regulatory network of the leaves, upregulated key genes associated with secondary metabolism and immune signaling pathways, and drove the production of antimicrobial compounds including flavonoids and hydroxy-sanshools at transcript levels. This ultimately helped establish a comprehensive defense system against leaf rust.

### 3.5. Expression Profiles of the DEGs and Metabolites in the Phenylpropanoid–Flavonoid Biosynthetic Pathway in S-YF and R-YF

**Figure 5:**
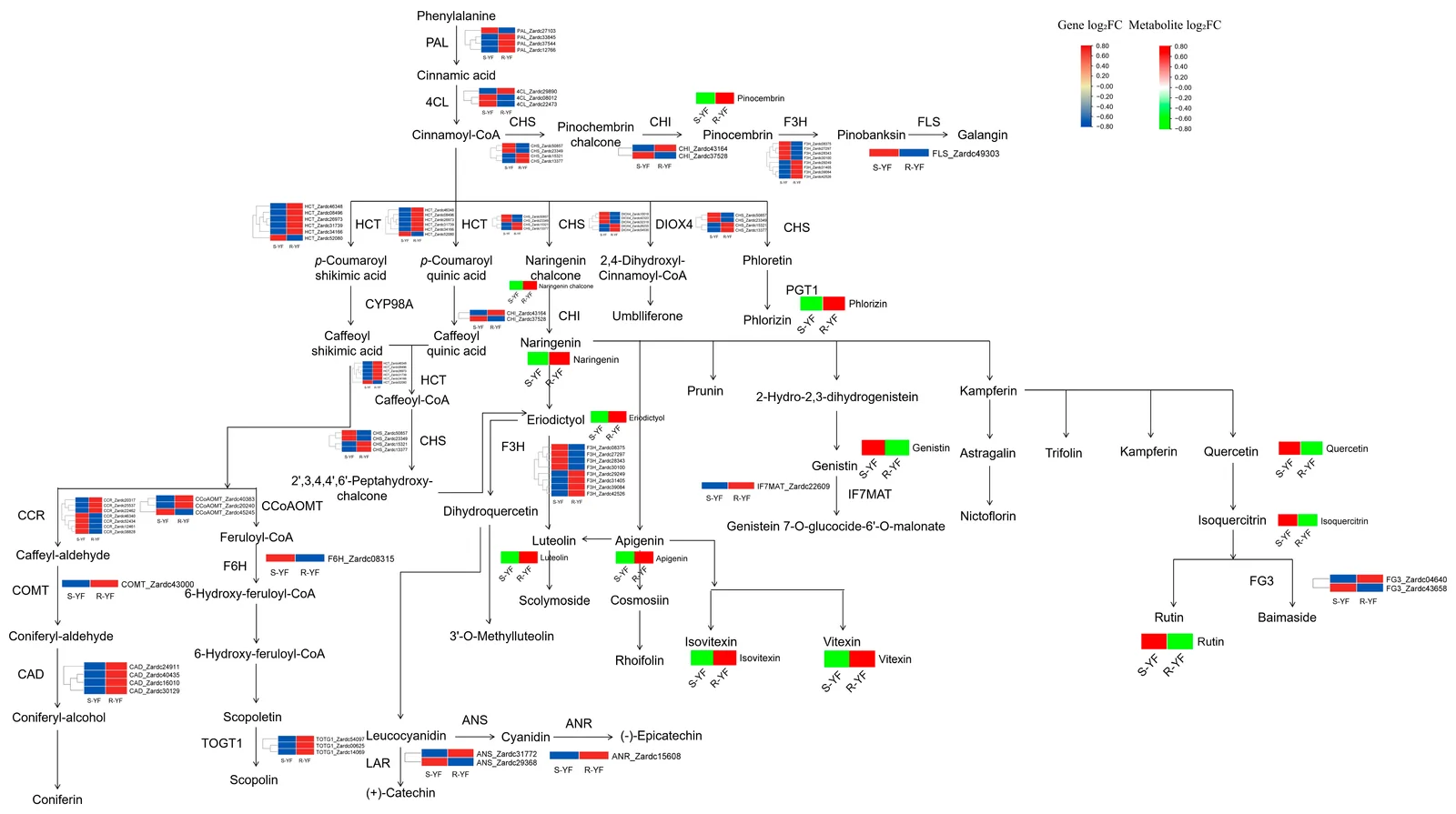
The differential expression map of the phenylpropanoid and flavonoid biosynthetic pathways in *Z. armatum*. The red–blue colour scale indicates gene expression changes (log_2_ fold change): red represents genes up-regulated in R-YF relative to S-YF, and blue represents genes with lower expression in R-YF relative to S-YF. The red–green colour scale indicates metabolite accumulation changes (log_2_ fold change): red represents metabolites with higher accumulation in R-YF, and green represents metabolites with lower accumulation in R-YF. Left, S-YF (rust-susceptible line); right, R-YF (rust-resistant bud sport line).

Flavonoids are key secondary metabolites in *Z. armatum*, exerting critical functions in shielding plants from diverse biotic and abiotic stressors. Combined multi-omics datasets uncovered that the DEGs and DAMs in S-YF and R-YF were significantly co-enriched in pathways related to flavonoid, flavonol, and phenylpropanoid biosynthesis. Phenylalanine served as the initial substrate in the upstream flavonoid biosynthetic pathway. It was catalyzed by *PAL* to produce cinnamic acid, which was then regulated by *4CL* and gradually branched into various flavonoid monomers, phenolic acids, and lignin precursors. Several key enzyme-coding genes were simultaneously annotated in the pathway, including *HCT*, *CHS*, *CHI*, *F3H*, flavonol synthase (*FLS*), feruloyl-CoA 6-hydroxylase (*F6H*), cinnamoyl-CoA reductase, caffeoyl-CoA O-methyltransferase (*CCoAOMT*), caffeic acid 3-O-methyltransferase (*COMT*), *CAD*, *TOGT1*, flavonol 3-O-glucosidase (*FG3*), isoflavone 7-O-glucoside-6″-O-malonyltransferase (*IF7MAT*), and *ANR*. The log_2_FC values and corresponding *p*-values for these key biosynthetic genes are provided in Table S2. Notably, Table S2 and Figure 5 include all annotated genes within this biosynthetic pathway and are not restricted to genes satisfying the transcriptome-wide DEG threshold |log_2_FC| ≥ 1, FDR < 0.05). Given that R-YF and S-YF are bud-sport derivatives originating from the same mother tree, modest transcriptional shifts for some pathway-associated genes are biologically plausible, and cumulative small-magnitude expression changes may contribute to downstream metabolic reprogramming.

Real-time quantitative polymerase chain reaction (RT-qPCR) analyses were performed for *4CL*, *HCT*, *F3H*, *CAD*, *TOGT1*, and *ANR*. These selected core rate-limiting genes exhibited statistically significant higher expression in R-YF (*p* < 0.05, *p* < 0.01, and *p* < 0.001) (Figure S5A–H), confirming consistency between RT-qPCR and transcriptome data. As visualized in the phenylpropanoid–flavonoid biosynthetic pathway diagram (Figure 5), the majority of core rate-limiting structural genes meeting our transcriptome-wide DEG criteria showed markedly higher expression in R-YF (red blocks in the red–blue gene scale), represented by red blocks according to the gene log_2_FC colour scale. Meanwhile, a small subset of pathway-related genes showed lower transcript abundance (blue blocks in the pathway map). These genes with lower transcript abundance mostly encode enzymes for minor secondary-branch modification, rather than core enzymes governing flavonoid backbone biosynthesis. Notably, this metabolic diagram only contains secondary-metabolism biosynthetic enzymes; canonical negative defense regulators and susceptibility-associated genes are not annotated within this biosynthetic pathway. Examination of our transcriptome data showed that such documented negative-regulator or susceptibility genes were not differentially expressed among these pathway-associated genes. Collectively, higher transcript levels of key enzyme genes fostered robust accumulation of various flavonoids in the R-YF leaves, such as apigenin, eriodictyol, isovitexin, luteolin, naringenin chalcone, naringenin, phlorizin, pinobanksin, and vitexin. Consistent trends between transcript and metabolite profiles demonstrated that broad activation of the flavonoid biosynthetic pathway constitutes a key molecular feature underlying leaf-rust resistance in the R-YF mutant.

At the metabolomic level, consistent with transcriptional activation of biosynthetic genes, most flavonoid-class DAMs assigned to this pathway displayed higher abundance in R-YF (red blocks in the red–green metabolite scale of Figure 5). These increased metabolites mainly belonged to flavone, flavonol and flavanone subclasses, including apigenin, eriodictyol, isovitexin, luteolin, naringenin chalcone, naringenin, phlorizin, pinobanksin, and vitexin. The log_2_FC values and *p*-values for these differentially accumulated flavonoids are listed in Table S3. Many of these compounds have previously been reported to possess antifungal and antioxidant activities, which may contribute to rust resistance in R-YF. In addition, a small number of phenylpropanoid-derived metabolites exhibited decreased accumulation in R-YF (green blocks in Figure 5). These metabolites with lower accumulation were mostly minor phenolic derivatives from side branches of the pathway and did not impair the overall enrichment of defense-related flavonoid compounds. Collectively, the coordinated increase in core biosynthetic gene expression and the enrichment of antifungal flavonoid metabolites reflected strong activation of the phenylpropanoid–flavonoid metabolic network.

### 3.6. Analysis of the Hydroxy-β-Sanshool Biosynthetic Pathway in S-YF and R-YF

**Figure 6:**
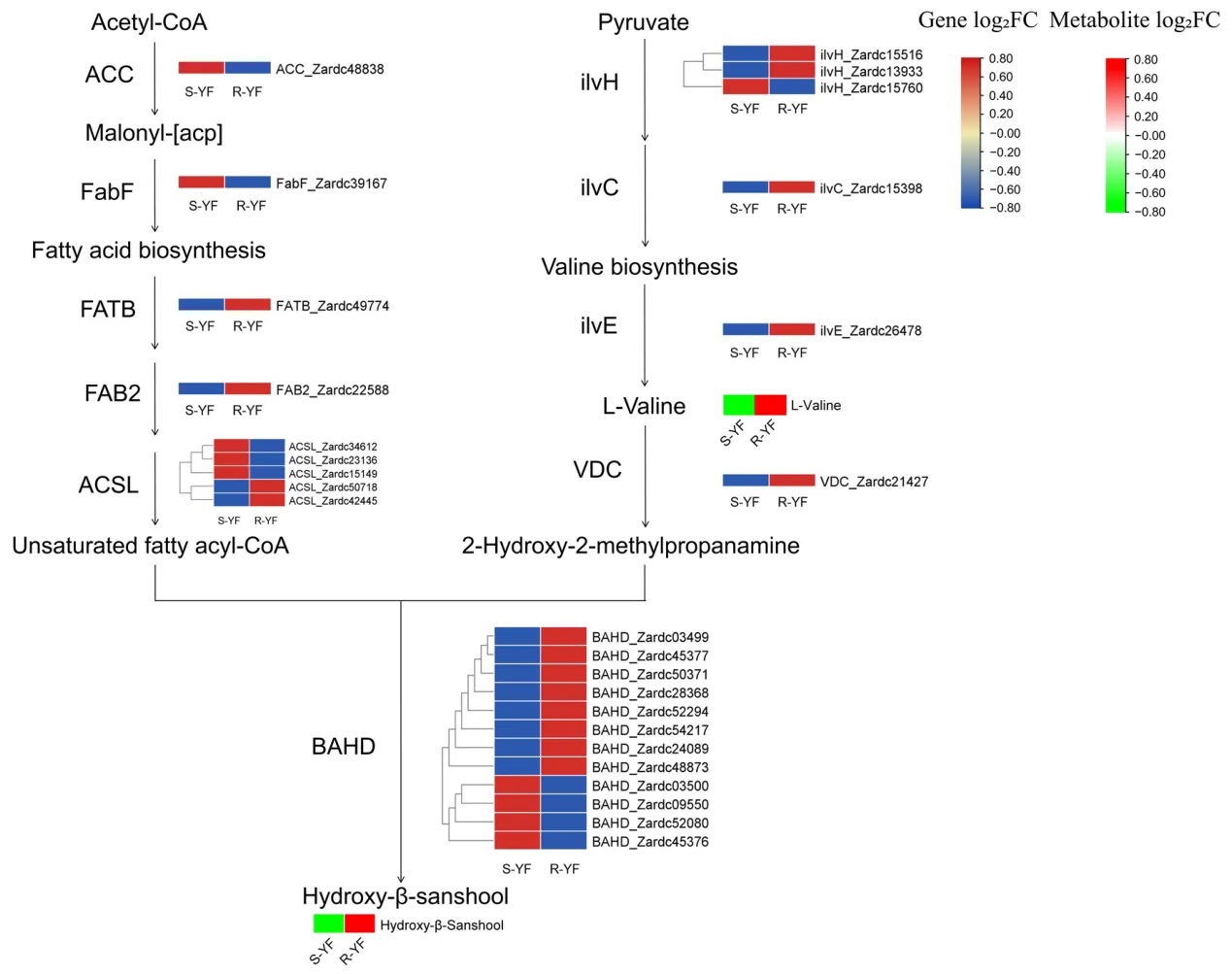
The precursor pathways for hydroxy-β-sanshool biosynthesis in *Z. armatum*. The left side shows the fatty acid biosynthetic pathway, while the right side displays the valine biosynthetic pathway. The two precursors are catalyzed by *BAHD* family enzymes to produce hydroxy-β-sanshool. The red–blue colour scale indicates gene expression changes (log_2_ fold change): red represents genes up-regulated in R-YF relative to S-YF, and blue represents genes with lower expression in R-YF relative to S-YF. The red–green colour scale indicates metabolite accumulation changes (log_2_ fold change): red represents metabolites with higher accumulation in R-YF, and green represents metabolites with lower accumulation in R-YF.

As shown in Figure 6, hydroxy-β-sanshool was synthesized via the condensation of two precursors, unsaturated fatty acyl-CoA and 2-hydroxy-2-methylpropanamine, catalyzed by *BAHD* acyltransferases. The unsaturated fatty acyl-CoA was derived from acetyl-CoA via a suite of fatty-acid biosynthetic genes: acetyl-CoA carboxylase (*ACC*), 3-oxoacyl-[acyl-carrier-protein] synthase II (*FabF*), fatty acyl-ACP thioesterase B (*FATB*), stearoyl-ACP desaturase (*FAB2*), and long-chain acyl-CoA synthetase (*ACSL*). The 2-hydroxy-2-methylpropanamine amine precursor was sequentially synthesized via genes linked to the valine metabolic pathway, including the acetolactate synthase small subunit (*ilvH*), ketol-acid reductoisomerase (*ilvC*), branched-chain amino acid transaminase (*ilvE*), and valine decarboxylase (*VDC*). The results showed that the *BAHD* genes responsible for final condensation exhibited higher expression in R-YF compared with S-YF (Figure S5I,J), while most of these genes showed relatively low expression in S-YF. The log_2_FC values and *p*-values for these *BAHD* and precursor pathway genes are provided in Table S2. As a result, the precursor L-valine and the defense metabolite hydroxy-β-sanshool accumulated in R-YF leaves. The log_2_FC values and *p*-values for L-valine and hydroxy-β-sanshool are listed in Table S3. These observations revealed that most *BAHD* family members showed markedly higher expression within R-YF, matching the abundant buildup of L-valine and hydroxy-β-sanshool. This suggests that the *BAHD*-mediated condensation reaction is a key step for hydroxy-β-sanshool biosynthesis and contributes to chemical-based rust resistance in R-YF.

### 3.7. Phylogenetic Classification, Chromosomal Distribution, and Physicochemical Properties of the ZaAP2/ERF Transcription Factor Family

Transcription factors exert essential functions in modulating secondary-metabolite biosynthesis and stress tolerance toward biotic and abiotic stimuli in plants. Statistical analysis of differentially expressed transcription factor gene families in the transcriptomes of the R-YF and S-YF leaves revealed that the annotated differentially expressed transcription factors belonged to various families, mainly including AP2/ERF, TIFY, bHLH, C2H2, MYB, and bZIP. The AP2/ERF family accounted for the highest proportion at 26.6%, followed by the TIFY family at 9.68%. This suggests that AP2/ERF members may serve as central modulators contributing to rust resistance within *Z. armatum* (Figure S6).

**Figure 7:**
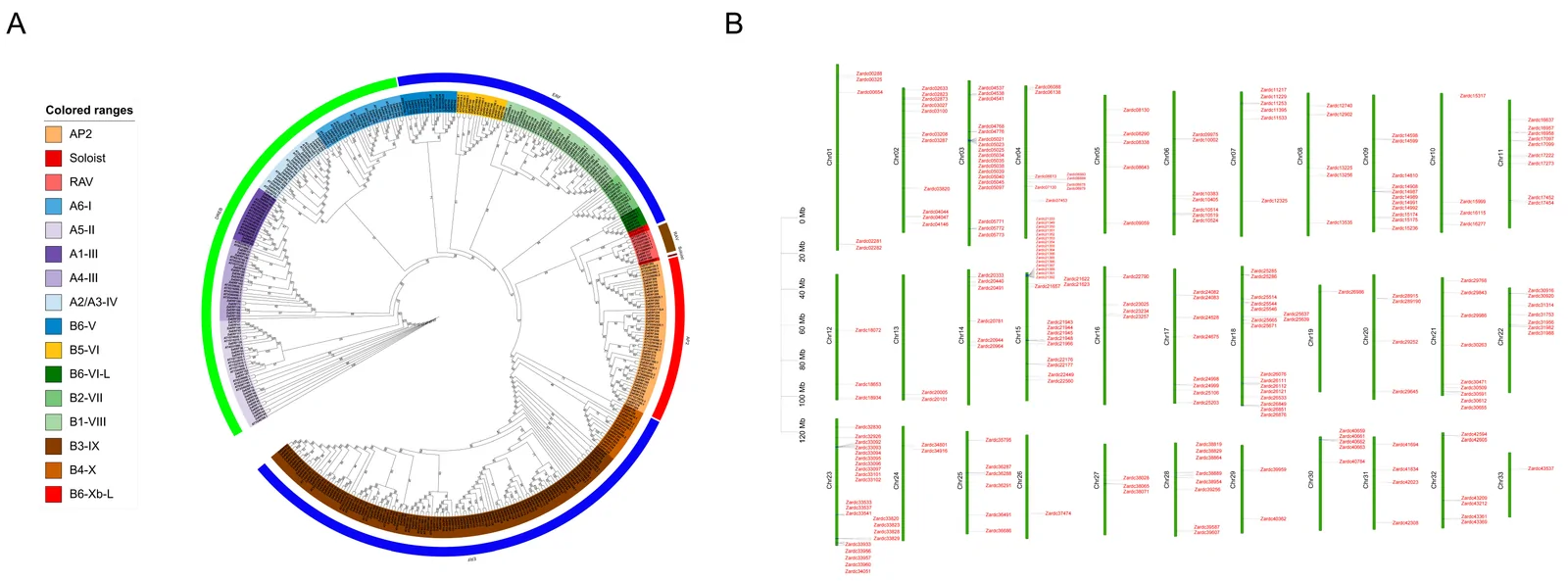
The phylogenetic tree and chromosomal distribution of the AP2/ERF transcription factor family in *Z. armatum*. (**A**) Circular phylogenetic dendrogram for the *ZaAP2/ERF* family. Different colored sectors represent different subfamilies. (**B**) The physical locations of the *ZaAP2/ERF* genes on the 33 chromosomes in *Z. armatum*. Green vertical lines indicate chromosomes, and red text labels gene IDs.

Table S4 presents the physicochemical properties of the *ZaAP2/ERF* family proteins. In total, 284 *ZaAP2/ERF* proteins were retrieved, with polypeptide lengths spanning 111–1020 amino acid residues. Their molecular weights ranged from 12.22 kDa to 113.54 kDa, while the isoelectric points varied from 4.00 to 10.57. Of these, 111 members with pI > 7 were basic proteins, while 173 with pI < 7 were acidic proteins. The instability index ranged from 27.15 to 84.30, with only 21 proteins classified as stable (index < 40), while the remaining 263 were unstable. The aliphatic index ranged from 38.75 to 89.39, indicating considerable variation in the thermal stability among family members. The grand average of hydropathy values was all negative, demonstrating hydrophilic nature for all *ZaAP2/ERF* proteins. Subcellular localization prediction showed that 226 members were localized in the nucleus, while the remaining 58 were distributed in the chloroplasts, mitochondria, cytoplasm, peroxisomes, extracellular space, and plasma membrane. A total of 284 *ZaAP2/ERF* genes were identified from the *Z. armatum* genome. A phylogenetic tree was constructed using Arabidopsis homologous sequences (Figure 7A), classifying the family into five major subfamilies: AP2, RAV, Soloist, DREB, and ERF. The DREB subfamily was further divided into six clades (A1–A6), and the ERF subfamily into seven clades (B1–B6 and B6-VI-L). The sequences in each clade were clustered tightly, indicating higher protein sequence conservation in the subfamilies. Chromosomal localization visualization (Figure 7B) showed that the 284 AP2/ERF genes were distributed across 33 chromosomes, with an overall dispersed pattern but several tandem gene clusters. Chromosome 3 exhibited the most pronounced tandem duplication enrichment, containing four gene clusters. These results indicated that the *ZaAP2/ERF* proteins exhibited a wide range of physicochemical properties, with the majority localized in the nucleus for transcriptional regulatory functions. The distinct phylogenetic clades and conserved sequence grouping illustrate the evolutionary connections inside this gene family. The presence of tandemly duplicated gene clusters on the chromosomes implies that tandem duplication acts as a major route driving family-scale gene expansion.

### 3.8. Sequence Analysis of the ZaAP2/ERF Transcription Factor Family

**Figure 8:**
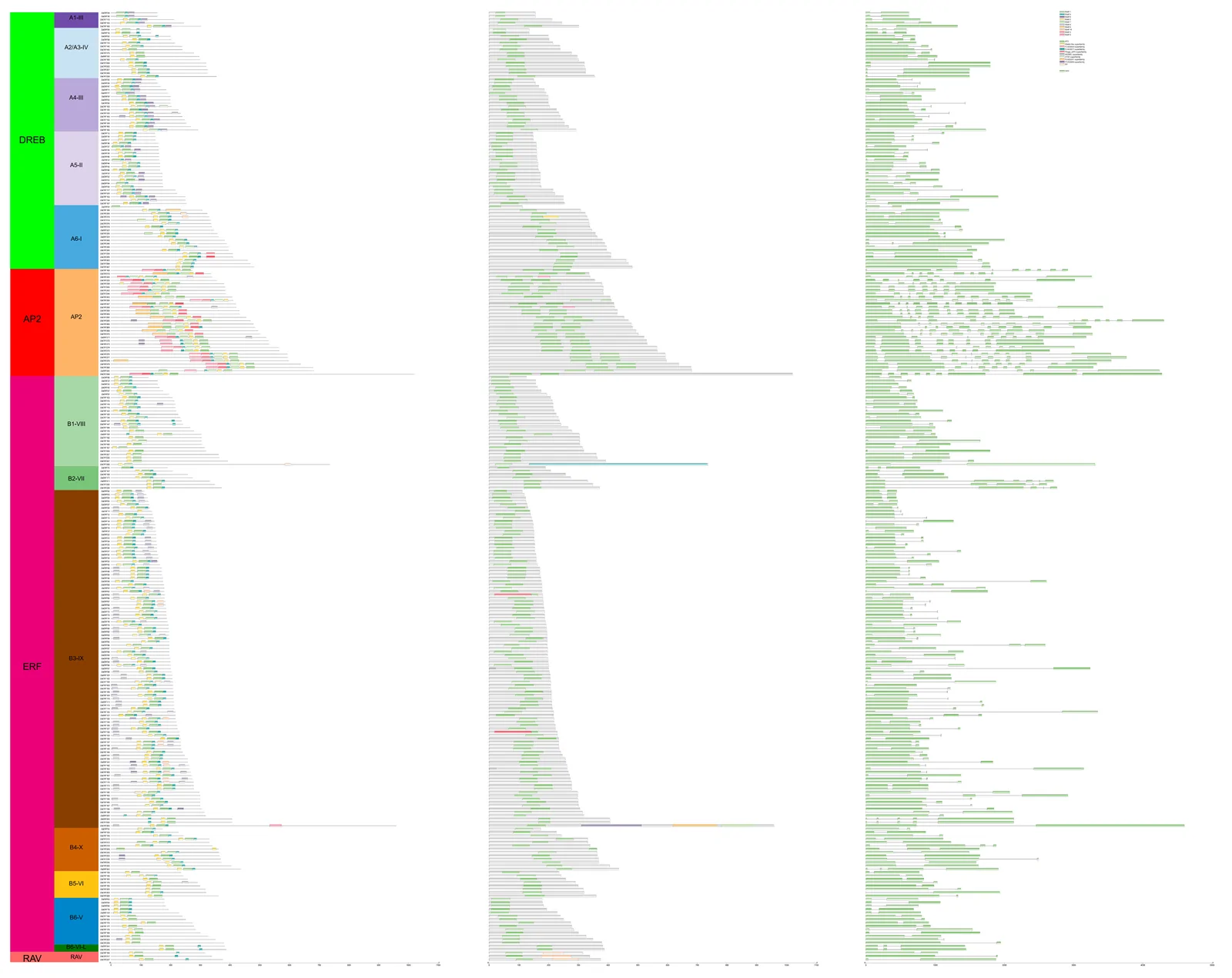
Integrated profiling of conserved motifs and gene architectures for *ZaAP2/ERF* transcription factors. Left: The phylogenetic tree showing subfamily and subgroup classification. The colored vertical bars represent different subfamilies and subgroups: green for DREB (including subgroups A1-III, A2/A3-IV, A4-III, A5-II, and A6-I); red for AP2; magenta for ERF (including subgroups B1-VIII, B2-VII, B3-IX, B4-X, B5-VI, B6-V, and B6-VI-L); and orange for RAV. Middle: The conserved protein motifs and CDD-annotated conserved functional domains. Right: The exon (green CDS) and intron gene organization.

A conserved-motif distribution profile for *ZaAP2/ERF* proteins was generated based on MEME conserved motif searches and CDD functional domain annotation. As shown in Figure 8, ten conserved motifs were detected across the 284 *ZaAP2/ERF* protein sequences. Motifs 1, 2, and 4 occurred in nearly all family members, marking the core conserved segments of this superfamily. The DREB subfamily contained 77 genes. The AP2 domains of the different subgroups consisted of specific motif combinations: those of subgroups A1–III, A4–III, and A5–II consisted of motifs 1, 2, 4, and 9, while those of subgroup A2/A3–IV comprised motifs 1, 2, and 4, and those of subgroup A6–I were characterized by motifs 1, 2, 4, and 7. A few DREB members carried unique motifs: ZaERF254 and ZaERF255 featured motif 5, ZaERF206 and ZaERF210 contained motif 10, and ZaERF196 was solely associated with motif 8. The AP2 subfamily comprised 32 genes, characterized by an AP2 domain consisting of motifs 1, 2, 3, 4, 5, 6, 7, and 9. The ERF subfamily, the largest with 172 genes, displayed motif composition divergence among clades. The AP2 domains of subgroups B1–VIII and B1–VII were composed of motifs 1, 2, and 4, with ZaERF118 and ZaERF147 also carrying motif 9, and ZaERF282 featuring motif 10, which corresponded to a PLN03077 superfamily conserved domain. The AP2 domain of subgroup B3-IX contained motifs 1, 2, 4, 6, 9, and 10, while ZaERF283 also featured motif 3 and was annotated with three superfamily domains: PTZ00009, PLN02241, and ATS1. The AP2 domains of subgroups B4-X, B5-VI, B6-V, and B6-VI-L consisted of motifs 1, 2, 4, 6, and 9. The RAV subfamily was represented by only three genes, which relied on motifs 1, 2, and 4 to form both the AP2 and B3 DNA-binding domains. Every one of the 284 protein sequences harbored a canonical AP2 conserved domain, verifying that these retrieved sequences belong to the AP2/ERF transcription factor superfamily. With only a few exceptions, AP2-subfamily proteins possessed two tandem-arranged AP2 domains. The ERF, DREB, and Soloist subfamily proteins retained only a single AP2 domain, while the RAV subfamily proteins possessed both AP2 and B3 DNA-binding domains. These structural features were consistent with the traditional classification criteria of the five major subfamilies. Gene structure analysis revealed that most of the DREB, ERF, and RAV subfamily members contained one intron and two exons. The AP2 subfamily exhibited more intricate gene architectures; most of its members harbored seven-to-eight introns corresponding to eight-to-nine exons. The conserved motif arrangement patterns tended to be consistent in the same phylogenetic clade, while significant variations in motif compositions across different subfamilies reflected closer evolutionary relationships and enhanced sequence conservation in the same subfamily.

### 3.9. Cis-Acting Regulatory Elements in the Promoters of the ZaAP2/ERF Genes

Transcription factors exert vital functions when plants cope with biotic and abiotic stress stimuli. The potential functions of the *ZaAP2/ERF* genes were explored by analyzing the regulatory cis-acting elements in their promoters (Figure S7). In total, 26 distinct cis-acting elements were detected, which fell into three major functional groups: environmental stress responses, hormone signaling responses, and growth and development regulation, totaling 5547 elements overall. The most prevalent among these were light-responsive elements, with 1391 elements (25.08% of the total), followed by 942 methyl jasmonate (MeJA)-responsive elements (16.98%) and 822 abscisic acid (ABA)-responsive elements (14.82%). The stress-related elements included those associated with anaerobic induction, low-temperature induction, drought-responsive MYB binding sites, wound response, and dehydration/low-temperature/salt stress. The hormone-responsive elements included MeJA, ABA, gibberellin, auxin, and salicylic acid. Additionally, various developmental elements were identified that regulated tissue differentiation, seed development, the cell cycle, and circadian rhythms, along with MYB binding sites involved in flavonoid biosynthesis regulation. These observations revealed that *ZaAP2/ERF* gene promoters harbor abundant cis-acting motifs linked to stress, hormone, and developmental processes. This implies that this gene family may simultaneously participate in hormone signal transduction, plant growth-related processes, and diverse defense responses against various stressors, including drought, low temperatures, and pathogen attacks, and may also modulate secondary flavonoid metabolic pathways.

### 3.10. Expression and RT-qPCR Validation of the ZaAP2/ERF Transcription Factor Genes

**Figure 9:**
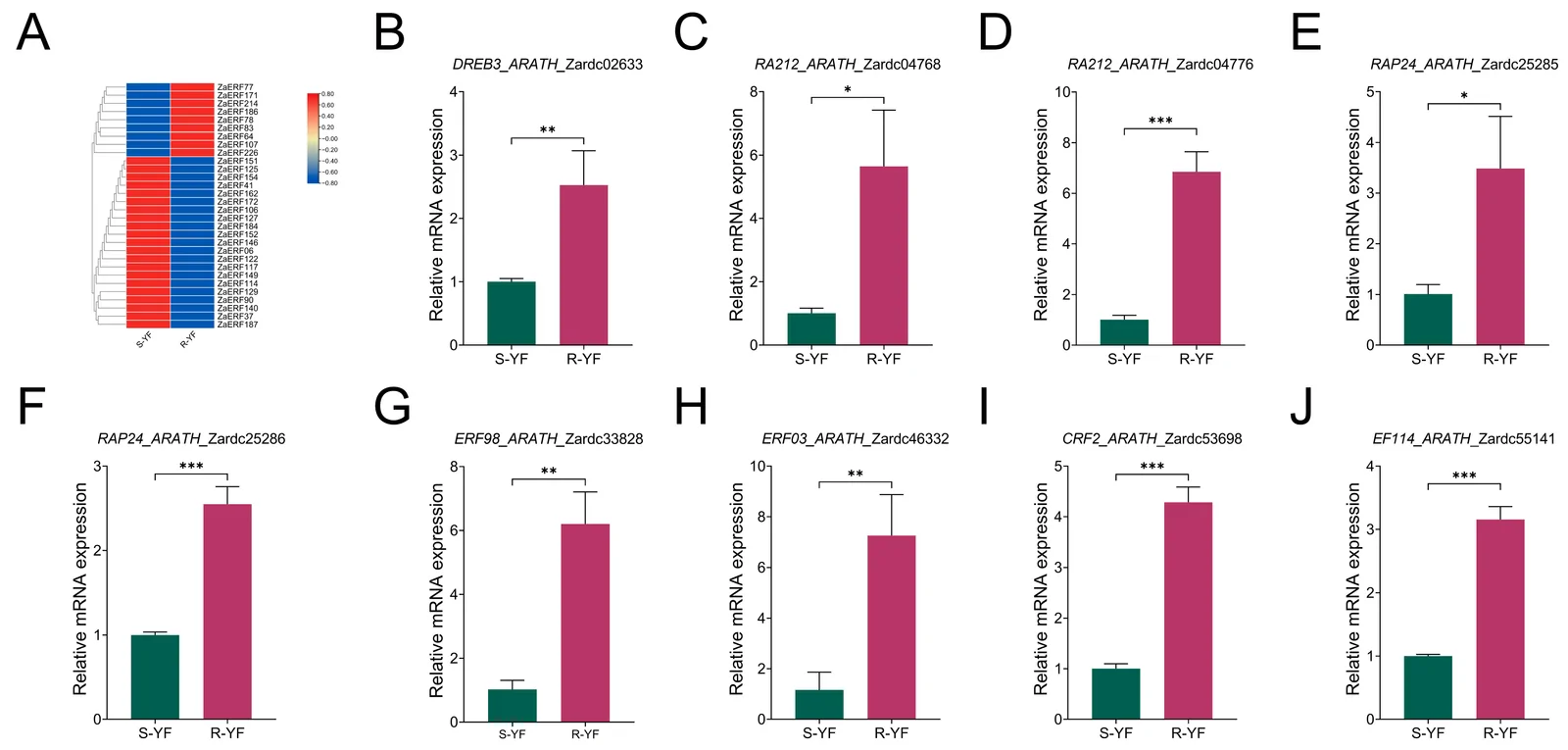
The differential expression and RT-qPCR validation of the *ZaAP2/ERF* transcription factors. (**A**) The expression heatmap of the *ZaAP2/ERF* genes in S-YF and R-YF. (**B**–**J**) The RT-qPCR quantification of nine significantly upregulated *ZaAP2/ERF* transcription factors. Data are presented as means ± SD (*n* = 3 biological replicates per group). * *p* < 0.05, ** *p* < 0.01, *** *p* < 0.001.

The 284 identified *ZaAP2/ERF* members were aligned to the full transcriptome gene set, yielding 232 matched genes for constructing an expression heatmap (Figure S8). These members were compared with the differentially expressed transcription factor dataset from the transcriptome, leading to the identification of 30 *ZaAP2/ERF* DEGs (Figure 9A). From these, nine significantly upregulated transcription factor-encoding genes in R-YF were selected for RT-qPCR validation. The heatmap illustrates the expression profiles of various *ZaAP2/ERF* genes in R-YF and S-YF. The RT-qPCR results for the nine selected upregulated *ZaAP2/ERF* transcription factors (Figure 9B–J) showed that the relative mRNA expression levels of all candidates were significantly higher in R-YF (*p* < 0.05, *p* < 0.01, and *p* < 0.001), and their expression trends were fully consistent with the transcriptome heatmap. These findings confirmed the reliability of the transcriptome sequencing data and indicated that these *ZaAP2/ERF* transcription factors are promising candidate genes putatively associated with the leaf-rust resistance response in *Z. armatum*.

## 4. Discussion

*Z. armatum* is an important woody plant valued for its culinary, medicinal, and economic properties [55]. However, leaf rust severely threatens the sustainable development of the industry, while the long-term reliance on chemical control can lead to environmental and health risks [56]. Therefore, it is essential to explore endogenous disease resistance mechanisms and utilize natural germplasm resources to promote eco-friendly disease management [57].

### 4.1. Physical and Chemical Defense Mechanisms

R-YF showed almost no visible rust infection symptoms at the physiological and phenotypic levels in natural field conditions, while its leaf lignin and total flavonoid content were significantly higher than in S-YF. Previous research has shown that lignin, a structural cell wall component, forms a physical barrier by thickening the epidermal and mesophyll cell walls. This impedes rust urediniospores attachment, hyphal penetration, and intercellular colonization. It plays a crucial role as a physical defense mechanism against fungal diseases in woody plants [58,59]. Flavonoid polyphenols not only directly inhibit rust spore germination and hyphal elongation but also scavenge the reactive oxygen species that accumulate during pathogenic infection, which alleviates cellular oxidative damage [60,61]. The HPLC quantification results of the present study showed significant accumulation of vitexin, rutin, and hydroxy sanshools in R-YF, suggesting that these compounds may function as potential endogenous antimicrobial metabolites in *Z. armatum*; however, their direct antimicrobial activity against *C. zanthoxyli* was not experimentally tested in this study. Furthermore, studies have shown that Woody plants such as black poplar and Norway spruce activate phenolic metabolism and SA/JA defense signaling upon rust infection, accompanied by elevated defense enzymes such as *PAL*, indicating a conserved role of flavonoids in woody plant rust responses [62,63]. Unlike these tree species, *Z. armatum* may also activate the hydroxy-sanshool biosynthetic pathway, creating a distinct chemical defense barrier via amide antimicrobial compounds. Although hydroxy sanshools are chemotaxonomic markers of *Zanthoxylum* [2,64], their defense function during the *Z. armatum*–rust interaction still requires further clarification through integrated metabolomic and transcriptomic analysis of both resistant and susceptible germplasm. Although the enhanced accumulation of lignin and flavonoids in R-YF supports the coordinated activation of physical and chemical defenses, additional defense-related biochemical markers, such as ROS accumulation, antioxidant enzyme activities, callose deposition, and endogenous phytohormone levels, were not directly measured in this study. Moreover, direct pathogen inhibition assays were not performed in this study; therefore, the antimicrobial activities of flavonoids and hydroxy sanshools against *C. zanthoxyli* remain to be experimentally verified. Future studies incorporating these assays will be necessary to further validate the proposed defense mechanisms and clarify the temporal dynamics of rust resistance in *Z. armatum.*

### 4.2. Multi-Omics Dissection of Defense Pathway Activation

Integrated transcriptomic and metabolomic analyses offer an effective way to understand the mechanisms underlying rust stress responses in woody plants [53]. *Z. armatum* is a heterozygous woody species with a large, complex genome shaped by a lineage-specific whole-genome duplication (WGD) and transposon expansion; this WGD expanded gene families related to flavonoid, branched-chain amino acid, and unsaturated fatty acid biosynthesis, providing a genetic basis for producing characteristic metabolites such as sanshools, flavonoids, and terpenoids [20]. The present study identified 1117 DEGs and 298 DAMs, which were co-enriched in the phenylpropanoid, flavonoid, and branched-chain amino acid degradation pathways, suggesting carbon and nitrogen reallocation toward defense-related secondary metabolites in resistant plants. Key flavonoid pathway genes, such as *PAL*, *4CL*, *HCT*, and *F3H*, were significantly upregulated in R-YF, which was associated with accumulation of antimicrobial flavonoids including naringenin, vitexin, and rutin, consistent with findings in *Z. bungeanum* cv. ‘Dahongpao’ [53]. The branched-chain amino acid degradation pathway supplies amine precursors for hydroxy-β-sanshool synthesis, while the unsaturated fatty acid biosynthetic pathway is simultaneously activated to provide acyl precursors. Together, these two pathways ensure the production of the unique *Z. armatum* amide defense compounds, a metabolic capability linked to the expansion of metabolism-related gene families driven by the lineage-specific WGD [20]. Furthermore, RT-qPCR results confirmed transcriptomic trends, supporting the synchronous activation of flavonoid and hydroxy-β-sanshool defense pathways. Beyond flavonoids and hydroxy-β-sanshools, other metabolite classes including amino acids and their derivatives, phenolic acids, lignans/coumarins, and terpenoids were also significantly altered. Branched-chain amino acid degradation may support nitrogen reallocation and energy metabolism; phenolic acids, lignans, and coumarins can reinforce cell walls and exert antimicrobial activity; and terpenoids may serve as antimicrobial or signaling molecules. Thus, metabolic reprogramming in R-YF involves coordinated shifts across multiple metabolite categories. Direct functional evidence for the contribution of these additional metabolites remains to be established.

From a temporal perspective, defense responses in R-YF can be organized into three layers: early defense signaling (hormone pathways and transcription factors such as *ZaAP2/ERF*), downstream metabolic reprogramming (phenylpropanoid–flavonoid and hydroxy-β-sanshool biosynthesis), and constitutive defense (higher basal lignin and flavonoid levels). Because samples were collected at a single field time point, this temporal hierarchy remains correlative and requires controlled time-course validation. In addition, natural field infection may introduce local variation in pathogen pressure, microclimate, and infection intensity; however, both lines originated from the same mother plant and were grown in the same plot, reducing large-scale environmental confounding. Controlled inoculation experiments are warranted to verify that the observed responses are specifically coupled to rust resistance.

### 4.3. ZaAP2/ERF Transcription Factors as Promising Candidate Regulators of Rust Resistance

The AP2/ERF family contained more DEGs than other transcription factor families, suggesting its role as a candidate upstream regulator in leaf-rust responses. AP2/ERF is a plant-specific superfamily comprising AP2, ERF, DREB, RAV, and Soloist subfamilies [65,66], widely involved in biotic stress, hormone signaling, and secondary metabolism regulation [51,67]. RAP2.6 (ERF subfamily) is significantly upregulated under salt stress in *Arabidopsis thaliana*, while its mutant exhibits enhanced sensitivity to salt stress, indicating that it positively regulates the salt stress response [68]. VvERF113, a root-enriched transcription factor, significantly improves the tolerance of grapevines to waterlogging stress by activating the hypoxia-responsive pathways [69]. The CRISPR/Cas9 knockout of *ZmEREB57* increased the sensitivity of maize to drought and salt stress, confirming its central role in adapting to multiple stress conditions [70]. An ethylene response element-binding factor identified in wild wheat enhanced the resistance to both biotic and abiotic stress [71]. Furthermore, AgDREB1 and AgDREB2 from celery significantly improve transgenic Arabidopsis tolerance to drought and low-temperature stress [72]. These studies collectively confirm the broad regulatory functions of the AP2/ERF family in plant defense and stress adaptation, providing experimental cross-species evidence that supports the putative inferred role of *ZaAP2/ERF* members in mediating the leaf rust response. Compared with herbaceous models, AP2/ERF studies in woody perennial rust pathosystems remain limited: in poplar, ERF genes are induced by Melampsora rust infection but their downstream targets are largely unknown; in pine, AP2/ERF members are associated with biotic stress signaling but have not been directly linked to secondary metabolite-based resistance. The present work extends these findings by connecting specific *ZaAP2/ERF* members to coordinated activation of flavonoid and hydroxy-β-sanshool biosynthesis, providing a novel link between this transcription factor family and species-specific antimicrobial amide metabolism. The present study identified 284 AP2/ERF-encoding genes based on the *Z. armatum* genome. Among the 284 identified *ZaAP2/ERF* members, only 30 were differentially expressed between R-YF and S-YF, among which nine were markedly upregulated in R-YF and selected for subsequent RT-qPCR validation. These candidate genes were prioritized because they displayed consistent upregulation in the resistant line, fell within phylogenetic subclades previously associated with plant biotic-stress responses, and harbored promoters enriched in stress- and hormone-responsive cis-elements. The remaining family members with negligible transcriptional responses may contribute to basal developmental regulation or abiotic stress adaptation or function redundantly with infection-induced homologs. Alternatively, some could be activated at later infection stages or in specific cell types that are not sufficiently captured by bulk-leaf transcriptome profiling. Accordingly, a lack of differential expression does not equal biological irrelevance, and leaf rust resistance in R-YF is likely orchestrated by a specific subset of *ZaAP2/ERF* family members. Chromosomal localization analysis showed that the family genes were widely distributed across all chromosomes, with tandem enrichment clusters in local regions. This suggests that both segmental duplication and species-specific WGD events have contributed to the expansion of this family. This evolutionary pattern is similar to that reported for the AP2/ERF family in *Z. bungeanum* cv. ‘Dahongpao’ [67]. Subcellular localization prediction revealed that most *ZaAP2/ERF* proteins were localized in the nucleus, which was consistent with the typical features of transcription factors that bind to target gene promoters to execute regulatory functions. This distribution pattern aligns with previous reports on the *ZaAP2/ERF* family [73]. Physicochemical parameter statistics indicated that the family proteins were mainly acidic, while all were hydrophilic. The conserved motif and gene structure analyses showed that the motif arrangement patterns of the *ZaAP2/ERF* proteins in the same phylogenetic clade were highly consistent. Motifs 1, 2, and 4 denoted core conserved segments shared by most family members, representing essential functional regions of the AP2 domain [67,74,75]. The ERF subfamily members in Zanthoxylum also participate in secondary terpenoid metabolism regulation [67]. Therefore, these transcription factors may potentially mediate the biosynthesis of multiple secondary antimicrobial metabolites, including flavonoids and sanshools, and could exert multifaceted regulatory roles in the defense against leaf rust in *Z. armatum*. The integration of the DEG screening and KEGG pathway enrichment results in this study showed that rust infection appeared to activate endogenous stress signaling pathways and significantly upregulate numerous *ZaAP2/ERF* genes. Together, these transcription factors may potentially modulate the transcription of downstream functional genes involved in flavonoid biosynthesis, hydroxy-β-sanshool production, and lignin deposition, which could contribute to antimicrobial metabolite accumulation and physical cell wall defense barrier construction. Therefore, *ZaAP2/ERF* proteins may represent promising candidate hubs that are proposed to link pathogenic stress-signal transduction to the initiation of downstream resistance-related metabolic processes.

**Figure 10:**
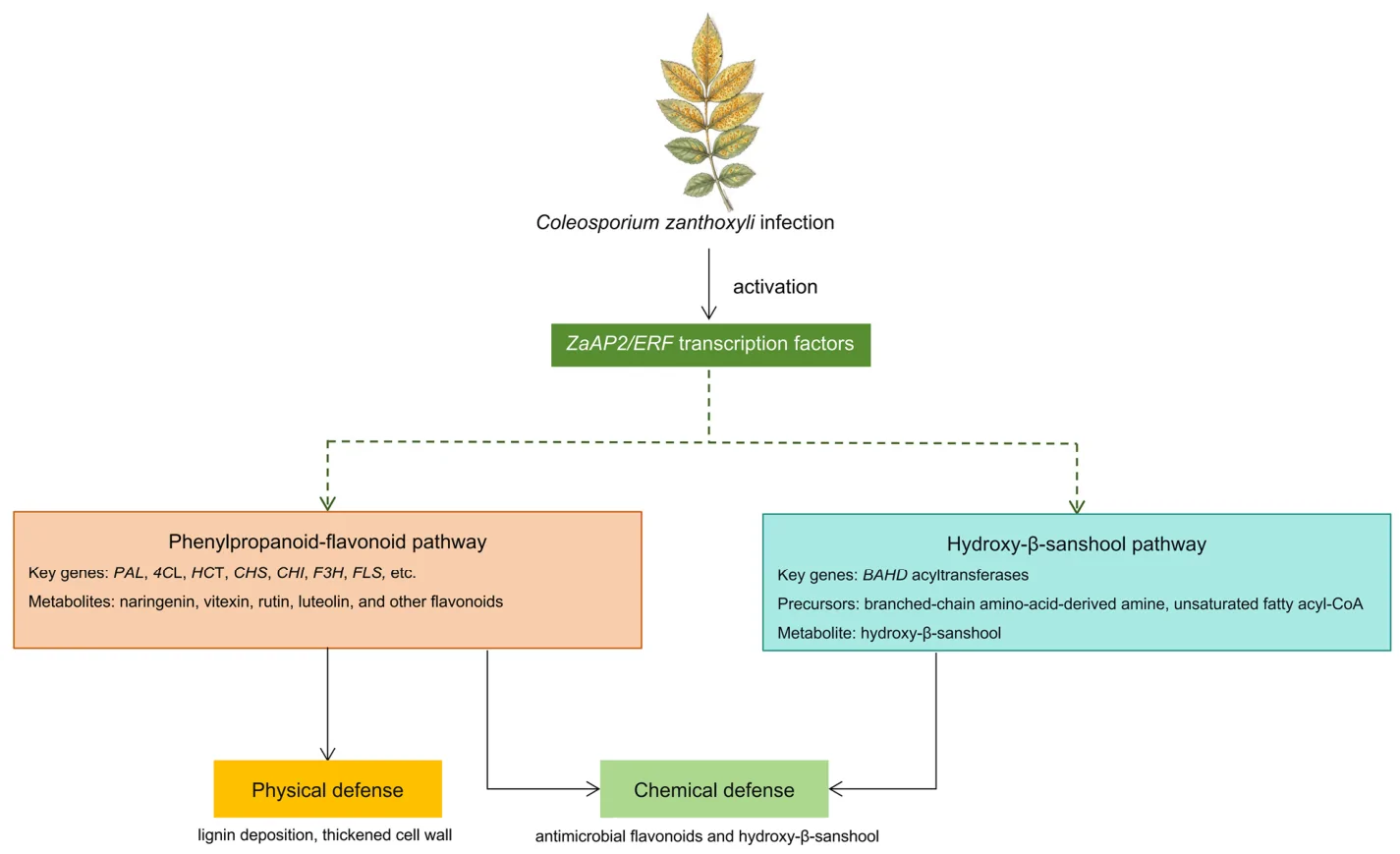
Proposed working model for leaf-rust resistance in *Z. armatum*. Infection by *Coleosporium zanthoxyli* triggers intracellular defense signaling in leaf cells. Multiple *ZaAP2/ERF* transcription factors are significantly up-regulated in resistant R-YF. Dashed arrows represent putative regulatory relationships inferred from co-expression and pathway enrichment, which remain to be verified by protein-binding assays. Solid arrows represent metabolic flow or observed physiological changes. These transcription-factor candidates may activate phenylpropanoid–flavonoid metabolism and hydroxy-β-sanshool biosynthesis. Elevated phenylpropanoid–flavonoid metabolism leads to lignin deposition for physical cell-wall reinforcement as well as flavonoid accumulation for chemical defense. Hydroxy-β-sanshool biosynthesis further contributes to chemical defense. The synergistic output of physical and chemical defenses confers strong leaf-rust resistance on R-YF.

Taken together, the integrated multi-omics evidence supports a working model in which rust infection triggers early-stage defense signaling, leading to transcriptional activation of specific *ZaAP2/ERF* genes. These transcription factors are proposed to coordinately regulate downstream phenylpropanoid–flavonoid and hydroxy-β-sanshool biosynthetic pathways. This regulatory cascade promotes lignin accumulation to reinforce physical cell-wall barriers, alongside enrichment of antimicrobial flavonoids and hydroxy-β-sanshool that constitute chemical defense. Combined activation of these physical and chemical defensive modules likely accounts for the enhanced leaf-rust resistance exhibited by the R-YF mutant. This hypothetical regulatory model is schematically summarized in Figure 10.

Several limitations of the present study should be acknowledged. First, all R-YF biological replicates were collected from spatially separated branches of a single bud-sport mutant rather than multiple genetically independent resistant individuals. Therefore, the current conclusions are based on a single mutant genotype, and future tests with additional resistant accessions are required to verify the conservation of these defense pathways. Second, although S-YF and R-YF originated from the same mother plant, whole-genome resequencing, SNP genotyping, or molecular marker validation was not performed. Thus, potential somatic genomic alterations unrelated to rust resistance cannot be fully excluded. Moreover, rust resistance was evaluated only by qualitative field symptoms. Quantitative disease assessment, spore germination inhibition assays, and controlled inoculation experiments are needed to rigorously confirm the resistant phenotype and to quantify the contribution of specific metabolites and genes. Third, the regulatory relationships between *ZaAP2/ERF* transcription factors and downstream biosynthetic genes were inferred from co-expression patterns and cis-element enrichment analysis, without direct physical binding or functional evidence. Fourth, the integration of transcriptomic and metabolomic data in this study was based primarily on pathway co-enrichment and RT-qPCR validation of representative genes. More comprehensive multi-omics integration approaches, such as WGCNA or regulatory network reconstruction, were not performed. Future studies applying these methods will be important to systematically identify regulatory relationships and further strengthen the mechanistic model. In addition, RT-qPCR validation was restricted to a limited set of representative genes. Future studies should expand the validation to more *ZaAP2/ERF* members and key structural genes, including *PAL*, *C4H*, *CHS*, *CHI,* and *FLS*. Finally, further biochemical and genetic experiments, such as gene overexpression, gene knockout, VIGS, yeast one-hybrid, EMSA, and dual-luciferase assays, are necessary to fully confirm the proposed regulatory network underlying leaf rust resistance in *Z. armatum.*

## 5. Conclusions

R-YF exhibited significantly stronger leaf rust resistance than S-YF. Upon rust infection, key genes within the flavonoid and hydroxy-β-sanshool biosynthetic pathways were synchronously up-regulated in R-YF, leading to markedly elevated levels of total flavonoids, lignin, and hydroxy-β-sanshool monomers. These flavonoids and hydroxy-β-sanshool metabolites likely suppress pathogen proliferation, whereas lignin deposition reinforces physical cell-wall barriers, suggesting synergistic action between chemical and physical defense responses. Multi-omics analysis revealed that differentially expressed genes and metabolites were predominantly enriched in flavonoid, flavonol and phenylpropanoid biosynthetic pathways. Among differentially expressed transcription factors, the AP2/ERF family showed the highest proportion. Of the 284 genome-wide-identified *ZaAP2/ERF* members, these transcription factors are proposed as potential candidate upstream regulators that may activate flavonoid and hydroxy-β-sanshool biosynthesis to mediate leaf rust resistance. Overall, flavonoid and hydroxy-β-sanshool biosynthetic pathways constitute core defense pathways against leaf rust, with *ZaAP2/ERF* transcription factors functioning as pivotal upstream activators. These findings provide a theoretical basis for disease-resistant germplasm breeding and sustainable leaf-rust management in *Z. armatum*.

## Data Availability

The raw data supporting the conclusions of this article will be made available by the authors on request.

## Supplementary Materials

The following supporting information can be downloaded at: https://www.mdpi.com/article/10.3390/biology15181638/s1. Figure S1: HPLC chromatograms of characteristic flavonoid and hydroxy sanshool metabolites in leaves of S-YF and R-YF. (A–F) Vitexin, hyperoside, rutin and isoquercitrin, hydroxy-α-sanshool, hydroxy-β-sanshool, and hydroxy-γ-sanshool, respectively. Gray traces represent standard compounds; red and blue traces represent S-YF and R-YF samples. Boxes indicate the peak positions of target components; Figure S2: KEGG functional annotation classification of differentially accumulated metabolites; Figure S3: KEGG functional annotation classification of differentially expressed genes; Figure S4: GO annotation classification of differentially expressed genes.; Figure S5: RT-qPCR quantification of key pathway genes. Beige bars represent S-YF (rust-susceptible line), and orange bars represent R-YF (rust-resistant bud-sport line). (A–F) Key flavonoid biosynthesis genes *HCT*, *4CL*, *TOGT1*, *CAD*, *F3H*, and *ANR*. (G–J) BAHD family genes involved in hydroxy-β-sanshool biosynthesis. * *p* < 0.05, ** *p* < 0.01, ** *p* < 0.001; Figure S6: Statistics of differentially expressed transcription factor gene families; Figure S7: Cis-acting regulatory elements in the promoters of *ZaAP2/ERF* genes; Figure S8: Heatmap of differential expression of 232 *ZaAP2/ERF* genes in leaves of the resistant line R-YF and the susceptible line S-YF. Color blocks represent gene expression levels as log_2_(fold change); red indicates up-regulation in R-YF, and blue indicates higher expression in S-YF; Table S1: Primer sequences; Table S2: Log_2_FC values of key genes involved in flavonoid and hydroxy-β-sanshool biosynthesis in R-YF compared with S-YF; Table S3: Differentially accumulated key flavonoids and hydroxy-sanshools in R-YF compared with S-YF; Table S4: Physicochemical properties and subcellular localization of *ZaAP2/ERF* family proteins in *Zanthoxylum armatum*.
