# Supplementary material for: Integrative Transcriptomic and Metabolomic Analysis Reveals the Molecular Regulatory Mechanisms of Leaf Rust Resistance in Zanthoxylum armatum
Source: Biology (Basel). 2026 Sep 16;15(18):1638. doi: 10.3390/biology15181638 (PMC13604301; doi:10.3390/biology15181638)
Supplement: Supplementary file 1 [file biology-15-01638-s001.zip › biology-4493648-supplementary.pdf]

Table S1 Primer sequences

Gene name	Primer sequences
Zardcβ-actin	F: TGGCCGTGATCTCACTGATG<br>R: CCATCAGGCAGCTCGTAGTT
Zardc02633	F: CGGAACTGGCTGACTCACTT<br>R: CCAAACTCGGCAACTCAACG
Zardc04768	F: TTCGTCACTAATCCCACCCG<br>R: AAAGGTGCCAAGCCAGACTC
Zardc04776	F: GGAAAGGGGTTCGAGTCTGG<br>R: CTTTCTTGCCGCGAATCTCG
Zardc25285	F: CCTTGTTGGCCTAGGAGGTG<br>R: GTACGTTAGCAGGGCCACAT
Zardc25286	F: TCTCAGCCCAAAACCTGTCTC<br>R: GTGCCTTTGTCTCACTCCCC
Zardc33828	F: GCTGAGATACGGGACCCTTC<br>R: CTCAAGTTAAACGCCGCACG
Zardc46332	F: CAGTTCTACCATTCGCCGGA<br>R: TCCTCGGGAACCCAATTTGT
Zardc53698	F: ACAACGCGGCTATTCAGCTA<br>R: ATTCGTCTCGCGTTTCTCGT
Zardc55141	F: ACAGAGGTGTACGGCAAAGG<br>R: AAGTGCCTAGCCAAACACGA
Zardc29890	F: GGTCTCACAAGCCGACGAAA<br>R: ACGCACAGGATCACATCTCG
Zardc15608	F: CTTGCGGCGAGCTTGATTAC<br>R: CAAATGTAGCGGCCAGAAGC
Zardc16010	F: GAGCACAAGCCTACCCCAAT<br>R: CTCCCGCAAAACGTTCACGA
Zardc25537	F: GTGGCATTAGCCCACATCCT<br>R: GTGACCCTTCTCCTGACAGC
Zardc54536	F: TACCCTTCATGCTGGGATGC<br>R: TCCTGATTGAGCGGTGATGT
Zardc31405	F: ACGCTCAGATGCACCTCTTC<br>R: TAAGCAACCCCGTCTTGGTC
Zardc42526	F: CTCGCCAGACAAGAACGAGT<br>R: CAAGGGTGACACAATTGGCG
Zardc08496	F: ACCGAGTGATCAAGCAAGCA<br>R: GGGGCTTTTGCAAGTGTGAG

Zardc54097	F: TGGGCACCACAGGTTTTGAT<br>R: TCACCCCCAGATCTTGACCA
Zardc00625	F: CTGCTGTTGCATGGCATTGT<br>R: ACACACCATGCCTTGAGGTT
Zardc03499	F: GAGCTTGCATCGCGTTCATT<br>R: TGTAGTAAGGATCACGCGCC
Zardc28368	F: AGAACGTGAAGGCACACGAT<br>R: CTCCTAATAGCCAGTGGCCG
Zardc50371	F: GCTCACCTCGTTGAATCGGA<br>R: AACTGTAGTCCCGAGACGGT
Zardc52294	F: AGGTTTCAGCAAAAGCTGCG<br>R: GCTTTCCCCATCCGAAGTCA
Zardc54217	F: TTGGCGATCTGCTGTTCGTA<br>R: CAGGCTTTCCCATGTGGCTA

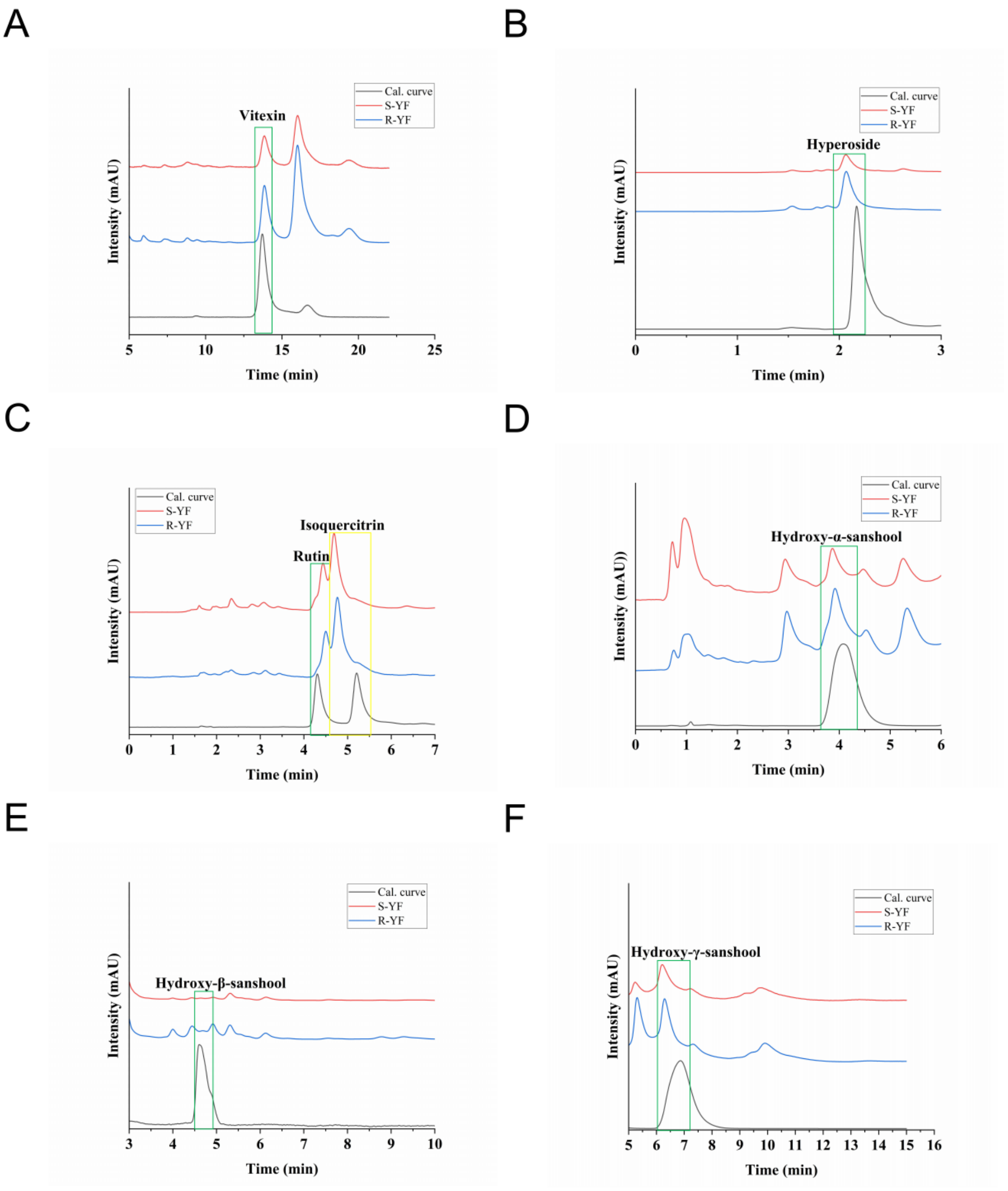

Figure S1. HPLC chromatograms of characteristic flavonoid and hydroxy sanshool metabolites in leaves of S-YF and R-YF. (A-F) Vitexin, hyperoside, rutin and isoquercitrin, hydroxy-α-sanshool, hydroxy-β-sanshool, and hydroxy-γ-sanshool, respectively. Gray traces represent standard compounds; red and blue traces represent S-YF and R-YF samples. Boxes indicate the peak positions of target components.

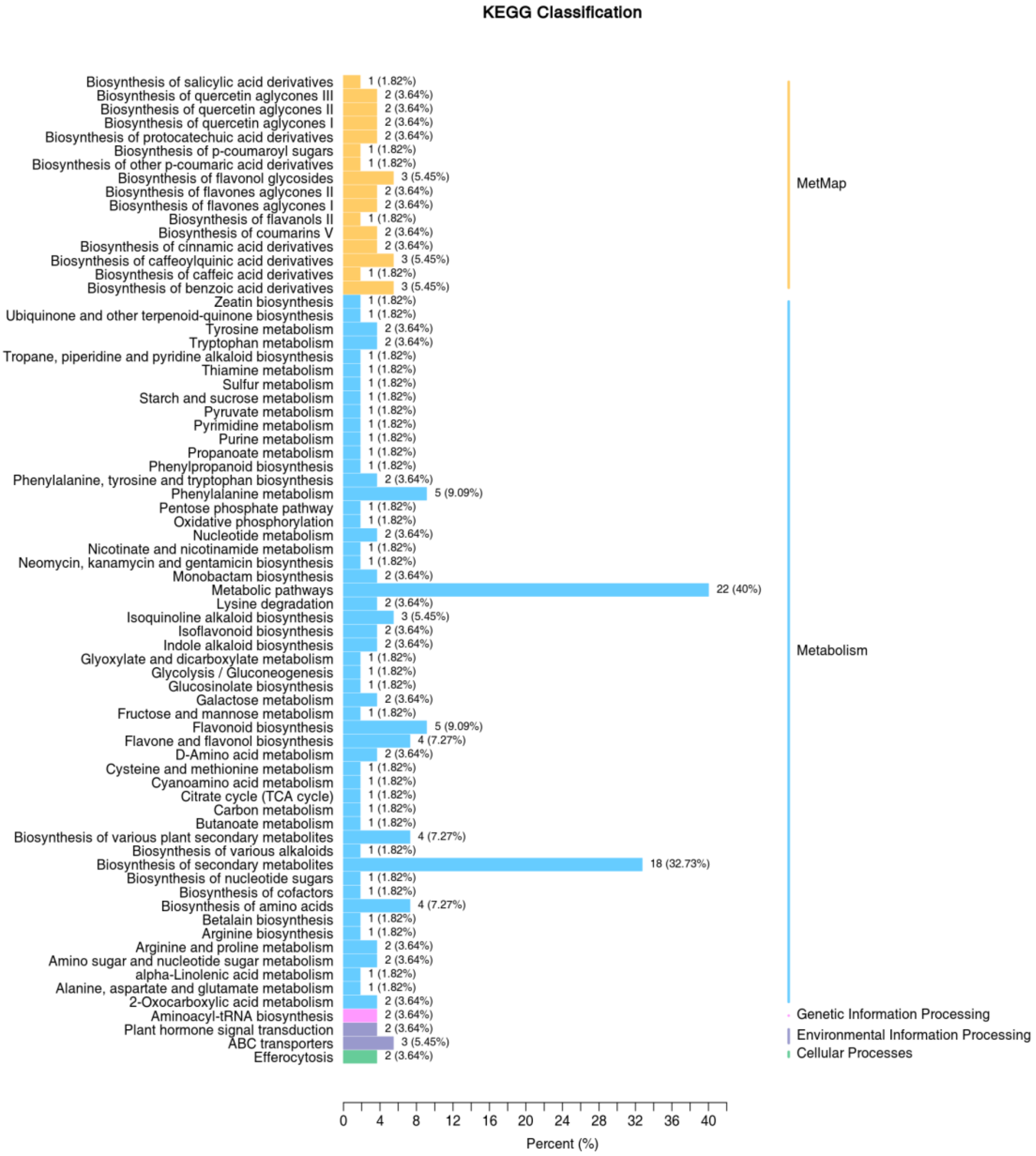

Figure S2. KEGG functional annotation classification of differentially accumulated metabolites.

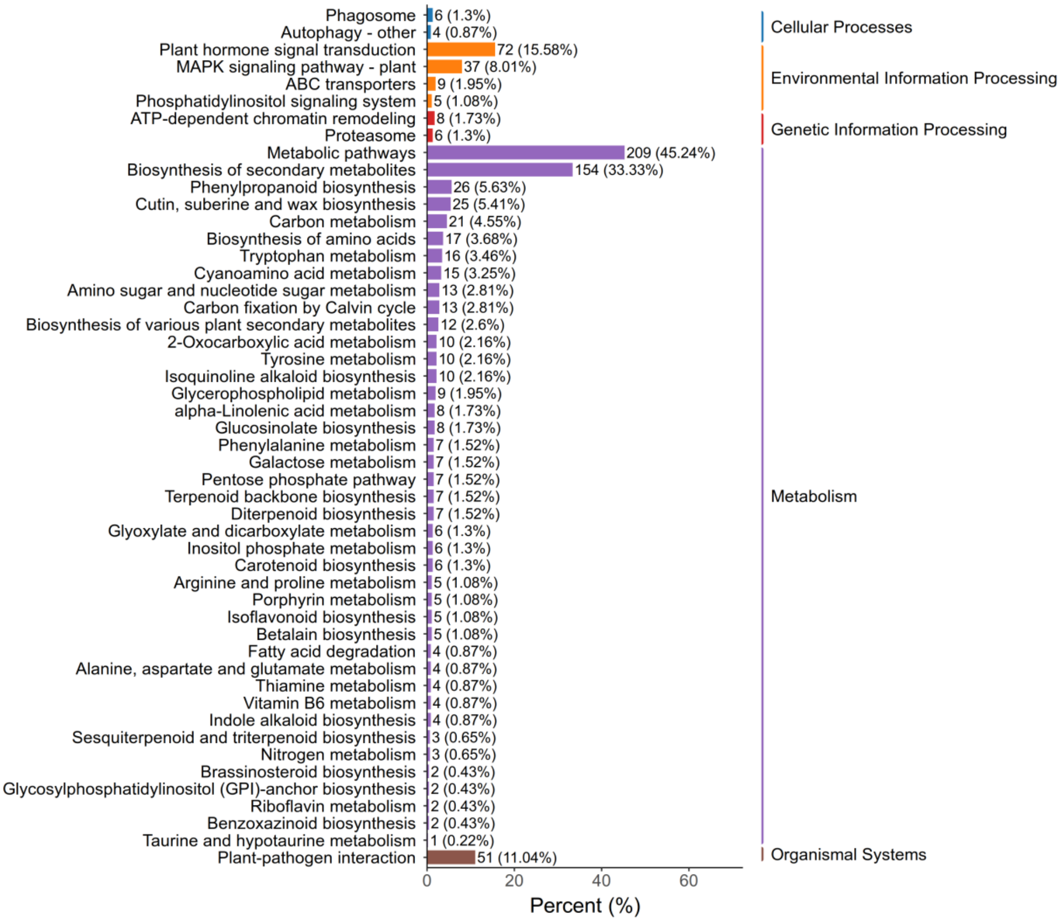

Figure S3. KEGG functional annotation classification of differentially expressed genes.

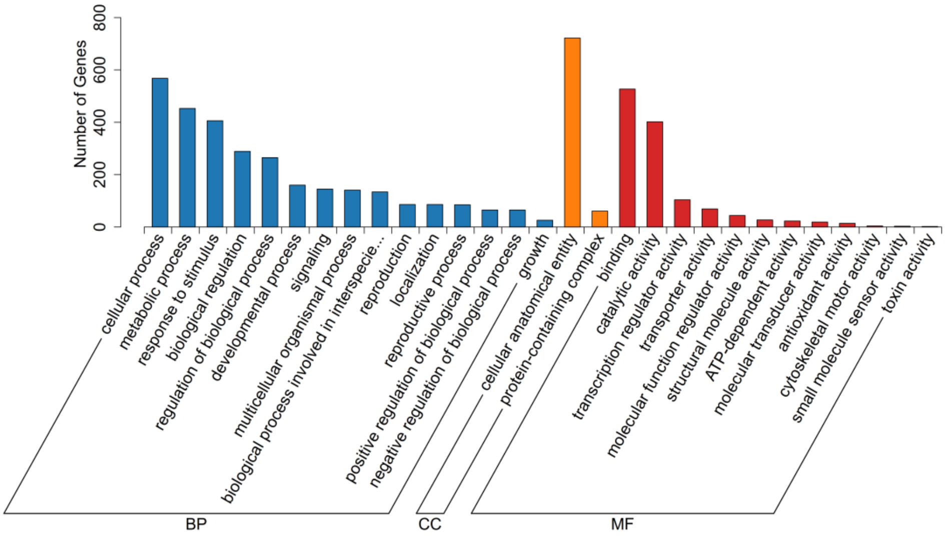

Figure S4. GO annotation classification of differentially expressed genes.

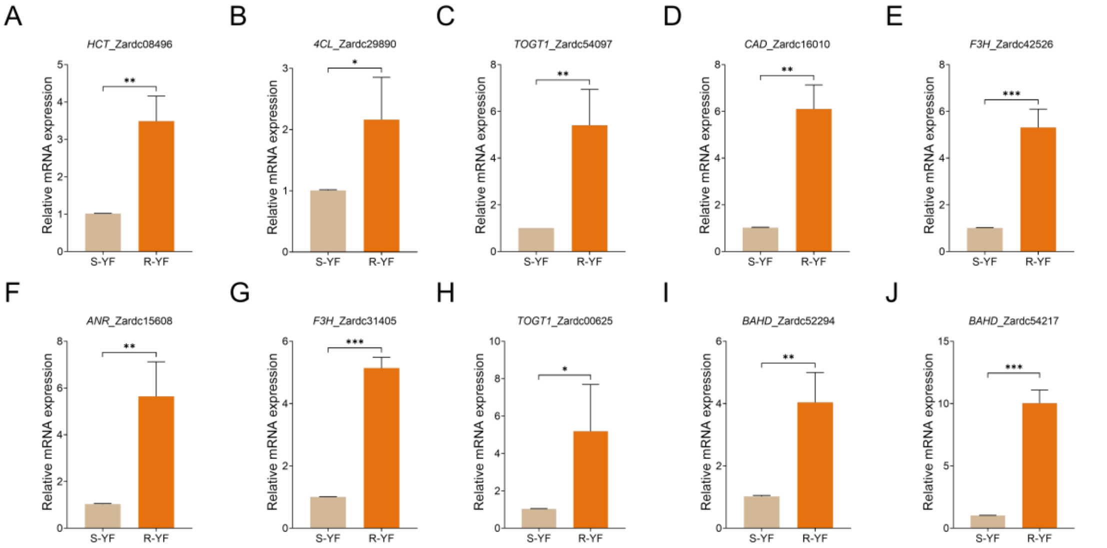

Figure S5. RT-qPCR quantification of key pathway genes. Beige bars represent S-YF (rust-susceptible line), and orange bars represent R-YF (rust-resistant bud-sport line). (A-F) Key flavonoid biosynthesis genes *HCT*, *4CL*, *TOGT1*, *CAD*, *F3H*, and *ANR*. (G-J) *BAHD* family genes involved in hydroxy-β-sanshool biosynthesis. *$P < 0.05$, **$P < 0.01$, **$P < 0.001$.

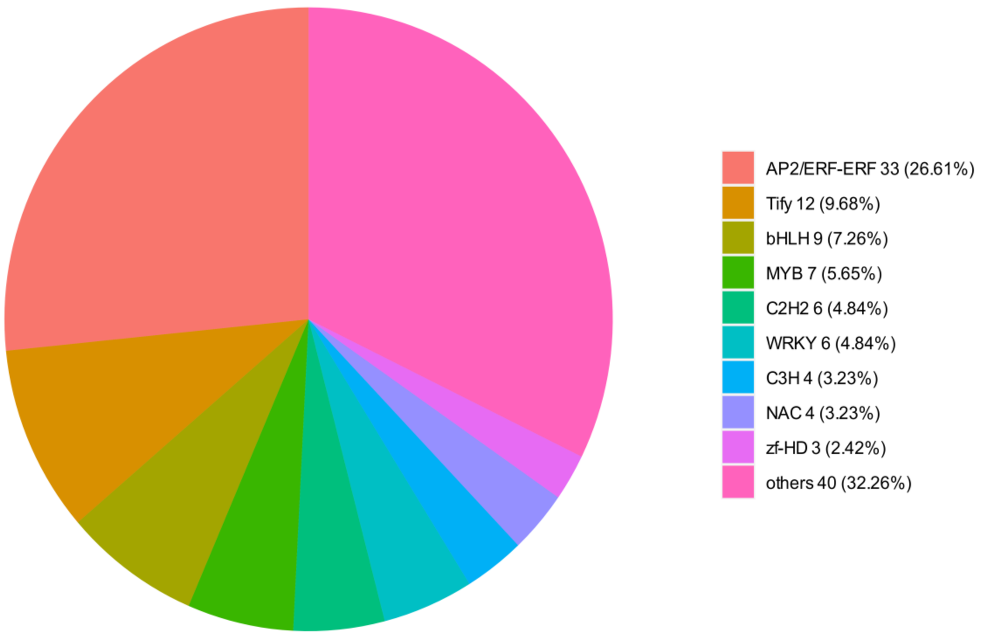

Figure S6. Statistics of differentially expressed transcription factor gene families

Table S2. Log_2FC values of key genes involved in flavonoid and hydroxy-β-sanshool biosynthesis in R-YF compared with S-YF

Gene ID	Gene Name	Log_2FC (R-YF/S-YF)	P-value
*PAL*_Zardc27103	*PAL*	-0.1032	0.3966
*PAL*_Zardc33845	*PAL*	0.47602	0.2673
*PAL*_Zardc37544	*PAL*	0.54612	0.2287
*PAL*_Zardc12766	*PAL*	0.14422	0.7588
*4CL*_Zardc29890	*4CL*	0.18252	0.2601
*4CL*_Zardc08012	*4CL*	-0.1140	0.3021
*4CL*_Zardc22473	*4CL*	-0.0523	0.3923
*CHS*_Zardc50857	*CHS*	-0.5747	0.1043
*CHS*_Zardc15321	*CHS*	0.1137	—
*CHS*_Zardc13377	*CHS*	—	—
*CHS*_Zardc23349	*CHS*	-0.0322	—
*CHI*_Zardc43164	*CHI*	0.0394	0.9334
*CHI*_Zardc37528	*CHI*	-0.0368	0.4481
*F3H*_Zardc08375	*F3H*	-0.1910	0.4251
*F3H*_Zardc27297	*F3H*	-0.0197	0.5553
*F3H*_Zardc28343	*F3H*	-0.4803	0.2152
*F3H*_Zardc29249	*F3H*	0.0931	0.7936
*F3H*_Zardc30100	*F3H*	-0.0029	0.5919
*F3H*_Zardc31405	*F3H*	0.8404	0.0230
*F3H*_Zardc39084	*F3H*	0.0903	0.8111
*F3H*_Zardc42526	*F3H*	0.0698	0.8840
*FLS*_Zardc49303	*FLS*	-0.9093	—
*HCT*_Zardc46348	*HCT*	0.2754	0.1447

*HCT*_Zardc08496	*HCT*	0.2107	0.2932
*HCT*_Zardc34166	*HCT*	0.9736	0.0094
*HCT*_Zardc26973	*HCT*	0.0583	—
*HCT*_Zardc31739	*HCT*	0.2908	—
*HCT*_Zardc52080	*HCT*	-0.0779	0.6867
*DIOX4*_Zardc10018	*DIOX4*	-3.1149	1.0022×10^-68
*DIOX4*_Zardc26255	*DIOX4*	0.1403	0.6684
*DIOX4*_Zardc54536	*DIOX4*	0.5380	0.1872
*DIOX4*_Zardc42320	*DIOX4*	-0.0685	0.2046
*DIOX4*_Zardc32318	*DIOX4*	-0.5188	2.6035×10^-5
*CCR*_Zardc20317	*CCR*	0.0410	0.8714
*CCR*_Zardc46340	*CCR*	-0.1600	0.0484
*CCR*_Zardc52434	*CCR*	-0.3270	0.0009
*CCR*_Zardc12461	*CCR*	-0.2202	—
*CCR*_Zardc25537	*CCR*	0.9579	4.5827×10^-16
*CCR*_Zardc38828	*CCR*	—	—
*CCR*_Zardc22462	*CCR*	0.4361	0.0119
*COMT*_Zardc43000	*COMT*	0.0341	0.8164
*CAD*_Zardc24911	*CAD*	0.0300	0.7347
*CAD*_Zardc40435	*CAD*	0.0965	0.9216
*CAD*_Zardc16010	*CAD*	0.3360	1.0074×10^-5
*CAD*_Zardc30129	*CAD*	0.0543	0.9576
*F6H*_Zardc08315	*F6H*	-0.4319	0.0495
*TOTG1*_Zardc54097	*TOTG1*	0.3689	0.0089
*TOTG1*_Zardc00625	*TOTG1*	0.5925	0.0058
*TOTG1*_Zardc14069	*TOTG1*	0.9119	—

*ANS*_Zardc31772	*ANS*	0.8880	—
*ANS*_Zardc29368	*ANS*	-0.2738	0.0094
*ANR*_Zardc15608	*ANR*	0.2049	0.4286
*IF7MAT*_Zardc22609	*IF7MAT*	0.2032	0.3161
*FG3*_Zardc04640	*FG3*	1.9028	—
*FG3*_Zardc43658	*FG3*	—	—
*CCoAOMT*_Zardc40383	*CCoAOMT*	0.0934	0.8987
*CCoAOMT*_Zardc45245	*CCoAOMT*	-0.1181	0.4266
*CCoAOMT*_Zardc20240	*CCoAOMT*	0.0354	0.8290
*BAHD*_Zardc03499	*BAHD*	0.1014	0.6235
*BAHD*_Zardc03500	*BAHD*	-0.0693	0.0407
*BAHD*_Zardc09550	*BAHD*	-0.3625	0.2759
*BAHD*_Zardc24089	*BAHD*	0.0593	0.9895
*BAHD*_Zardc28368	*BAHD*	0.0303	0.8816
*BAHD*_Zardc45376	*BAHD*	-0.6684	0.0090
*BAHD*_Zardc45377	*BAHD*	0.0215	0.7319
*BAHD*_Zardc48873	*BAHD*	0.0523	0.9801
*BAHD*_Zardc50371	*BAHD*	0.1256	0.4155
*BAHD*_Zardc52080	*BAHD*	-0.0779	0.6867
*BAHD*_Zardc52294	*BAHD*	0.6799	1.3215×10^-7
*BAHD*_Zardc54217	*BAHD*	0.2837	0.0578
*ACC*_Zardc48838	*ACC*	-0.3800	0.4634
*ACSL*_Zardc34612	*ACSL*	-0.5280	1.1088×10^-6
*ACSL*_Zardc50718	*ACSL*	0.092816	0.8720
*ACSL*_Zardc15149	*ACSL*	-0.018482	0.7111
*ACSL*_Zardc42445	*ACSL*	0.064743	0.9111

*ACSL*_Zardc23136	*ACSL*	-1.028828	2.0494×10^-23
*ADC*_Zardc11934	*ADC*	0.316305	0.0001
*ADC*_Zardc02915	*ADC*	0.633039	—
*FAB2*_Zardc22588	*FAB2*	0.347545	0.0030
*FabF*_Zardc39167	*FabF*	-0.045210	0.2712
*FAD3*_Zardc29372	*FAD3*	-0.780362	5.3927×10^-8
*FATB*_Zardc49774	*FATB*	0.170886	0.2285
*GNAT*_Zardc37495	*GNAT*	0.065448	0.9287
*GNAT*_Zardc10588	*GNAT*	0.161768	0.4606
*GNAT*_Zardc37538	*GNAT*	0.410978	0.1137
*ilvC*_Zardc15398	*ilvC*	0.266903	0.0017
*ilvE*_Zardc26478	*ilvE*	0.563354	4.3849×10^-6
*ilvH*_Zardc15516	*ilvH*	0.106302	0.8182
*ilvH*_Zardc15760	*ilvH*	-0.121585	0.2130
*ilvH*_Zardc13933	*ilvH*	0.115709	0.4407
*LDC*_Zardc06843	*LDC*	0.341668	0.0238
*LDC*_Zardc26580	*LDC*	-0.248557	0.0578
*LDC*_Zardc49460	*LDC*	0.219589	0.4659
*PYDC*_Zardc24700	*PYDC*	-0.000398	0.4904
*SLD*_Zardc14838	*SLD*	0.051628	—
*VDC*_Zardc21427	*VDC*	0.085416	0.8647

Note: This table includes all annotated pathway-related structural genes, not limited to transcriptome-wide differentially expressed genes defined by $|log_2FC| \geqslant 1$ *and* $FDR < 0.05$.

Table S3. Differentially accumulated key flavonoids and hydroxy-sanshools in R-YF compared with S-YF

Metabolite name	Log_2FC (R-YF/S-YF)	P-value
Rutin	-0.4294	—
Apigenin	0.3743	—
Eriodictyol	1.3699	0.1963
Genistin	-0.2829	—
Hydroxy-β-Sanshool	0.3924	—
Isoquercitrin	—	—
Isovitexin	0.3977	—
Luteolin	0.2219	—
L-Valine	0.1427	—
Naringenin Chalcone	1.0391	0.0197
Naringenin	0.6209	—
Phlorizin	0.1612	—
Pinocembrin	0.5364	—
Quercetin	-2.9069	0.0704
Vitexin	—	—

Table S4. Physicochemical properties and subcellular localization of *ZaAP2/ERF* family proteins in *Zanthoxylum armatum.*

New ID	Number of Amino Acid	Molecular Weight	Theoretical pI	Instability Index	Aliphatic Index	Grand Average of Hydropathicity	Subcellular localization
ZaERF273	531	58794.29	5.76	51.59	59.6	-0.712	nucl
ZaERF272	530	58722.25	5.78	50.4	58.79	-0.72	nucl
ZaERF183	298	32715.76	6.45	67.72	55.07	-0.747	nucl
ZaERF59	175	20155.95	9.93	51.94	66.97	-0.906	chlo
ZaERF185	299	33504.47	5.11	46.77	63.71	-0.56	nucl

ZaERF77	191	21144.67	4.85	41.35	80.79	-0.267	nucl
ZaERF75	189	20897.4	5	58.04	58.89	-0.641	nucl
ZaERF192	304	34060.19	5.5	49.58	46.61	-0.84	nucl
ZaERF121	216	24100.74	9.47	73.13	57.96	-0.809	nucl
ZaERF248	394	44020.63	9.27	50.96	73.55	-0.648	chlo
ZaERF151	246	27855.3	9.01	47.32	66.26	-0.772	nucl
ZaERF228	360	39746.58	4.79	54.53	59.36	-0.605	nucl
ZaERF188	300	33106.93	6.08	46.78	68.33	-0.659	nucl
ZaERF254	409	45383.98	9.15	50.5	72.76	-0.629	nucl
ZaERF255	409	45383.98	9.15	50.5	72.76	-0.629	nucl
ZaERF25	153	17211.99	5.99	62.59	54.9	-0.967	nucl
ZaERF168	273	30260.51	6.26	50.36	62.89	-0.658	nucl
ZaERF76	189	20967.35	9.47	49.37	61.06	-0.73	nucl
ZaERF18	148	16579.98	5.64	66.55	53.58	-0.839	nucl
ZaERF171	274	30993.72	6.78	46.59	66.28	-0.79	nucl
ZaERF107	207	23132.07	9.62	37.29	64.15	-0.743	nucl
ZaERF84	195	22115.82	7.83	44.41	69.13	-0.726	nucl
ZaERF174	277	30841.26	5.08	58.33	61.34	-0.696	nucl
ZaERF110	209	23350.74	5.84	40.57	55.6	-0.725	nucl
ZaERF126	222	24667.29	5.34	68.03	65.5	-0.648	nucl
ZaERF21	150	17196.2	9.8	84.3	60.53	-1.077	nucl
ZaERF108	209	23380.82	5.84	40.57	56.08	-0.708	nucl
ZaERF173	277	30852.22	5.08	58.95	61.7	-0.717	nucl
ZaERF133	230	26077.06	7.03	52.5	65.39	-0.866	nucl
ZaERF180	292	31811.56	5.55	58.09	54.97	-0.813	nucl
ZaERF178	284	30817.51	9.72	48.57	64.86	-0.566	cyto

ZaERF96	200	22336.13	9.89	49.37	67.45	-0.717	nucl
ZaERF88	196	22026.7	9.94	54.07	63.32	-0.849	nucl
ZaERF61	178	20032.23	5.6	60.28	64.27	-0.562	nucl
ZaERF79	193	21676.67	9.17	52.65	67.82	-0.64	chlo
ZaERF131	228	25571.49	8.27	57.48	67.59	-0.834	nucl
ZaERF141	236	26130.99	9.12	51.56	55.89	-0.777	nucl
ZaERF125	221	24452.47	5.06	50.82	73.3	-0.343	cyto
ZaERF20	149	16211.96	9.72	37.93	56.98	-0.745	cyto
ZaERF154	249	27758.12	4.43	64.65	58.8	-0.774	cyto
ZaERF52	172	19338.25	4.51	54.65	49.42	-0.807	cyto
ZaERF274	564	61503.36	6.58	49.12	59.24	-0.627	nucl
ZaERF43	162	17862.69	7.84	60.48	54.26	-0.919	nucl
ZaERF177	278	31204.84	8.39	45.21	62.09	-0.715	chlo
ZaERF205	320	35577.87	5.78	42.34	70.78	-0.603	nucl
ZaERF117	215	23684.44	4.25	49.63	55.4	-0.907	cyto
ZaERF204	320	35716.11	6.17	43.44	67.44	-0.621	nucl
ZaERF218	338	38001.66	6.48	42.51	66.12	-0.627	nucl
ZaERF219	339	38505.08	5.53	50.14	61.39	-0.901	nucl
ZaERF175	277	29293.2	9.32	48.94	57.51	-0.575	nucl
ZaERF237	375	41941.16	8.74	41.1	68.85	-0.599	nucl
ZaERF245	387	42545.7	5.12	49.54	75.87	-0.306	chlo
ZaERF197	314	35258.91	6.45	52.44	54.71	-0.742	nucl
ZaERF227	360	40096.23	6.79	60.17	51.25	-0.801	nucl
ZaERF232	363	40496.58	6.6	59.55	49.75	-0.826	nucl
ZaERF210	329	36357.84	8.08	61.47	67.42	-0.583	nucl
ZaERF206	323	35483.43	5.34	59.96	69.26	-0.581	nucl

ZaERF157	253	27484.7	5.33	66.85	70.28	-0.254	mito
ZaERF235	369	40296.42	6.67	69.24	59.86	-0.666	nucl
ZaERF54	172	19219.79	8.88	51.73	71.57	-0.491	nucl
ZaERF190	301	32020.52	5.2	56.99	70.8	-0.437	chlo
ZaERF268	483	53268.08	5.64	60.01	59.61	-0.62	nucl
ZaERF104	207	23833.86	5.74	52.14	81.5	-0.568	nucl
ZaERF164	265	29725.35	4.94	75.52	45.32	-0.849	nucl
ZaERF86	195	21768.63	5.76	64.59	40.67	-1.029	nucl
ZaERF129	226	25610.59	9.48	56.33	49.78	-1.016	chlo
ZaERF24	152	16739.33	6.31	53.99	51.51	-0.802	nucl
ZaERF05	124	14162.53	4.83	49.77	55.97	-0.955	mito
ZaERF139	235	27148.46	4.77	53.63	54.34	-0.98	nucl
ZaERF144	239	25878.68	9.3	51.31	60.46	-0.594	chlo
ZaERF167	270	30131.15	5.13	57.58	67.19	-0.699	nucl
ZaERF136	233	26065.28	5.87	33.82	74.55	-0.437	cyto
ZaERF94	199	21781.07	5.69	67.9	64.72	-0.696	nucl
ZaERF158	256	28289.59	6.09	58.93	71.25	-0.522	nucl
ZaERF23	151	16652.26	6.31	53.01	51.85	-0.802	nucl
ZaERF14	145	16155.71	4.88	63.02	57.93	-0.692	nucl
ZaERF147	241	26227.19	8.89	59.62	60.08	-0.609	nucl
ZaERF193	304	33649.83	5.62	61.37	46.91	-0.753	nucl
ZaERF81	193	20516.73	8.79	52.9	59.69	-0.645	nucl
ZaERF264	468	52160.79	6.08	54.7	62.78	-0.738	nucl
ZaERF119	215	23299.29	8.86	57.84	61.3	-0.485	nucl
ZaERF39	160	17687.14	10.57	56.56	70.88	-0.646	nucl
ZaERF234	368	39733.6	7.94	57.92	49.18	-0.65	nucl

ZaERF233	364	39221.92	8.77	61.23	46.51	-0.712	nucl
ZaERF240	381	42304.99	5.89	62.38	66.64	-0.715	nucl
ZaERF246	388	43355.23	6.03	59.42	64.41	-0.784	nucl
ZaERF16	148	16683.87	9.86	50.29	62.77	-0.824	nucl
ZaERF17	148	16683.87	9.86	50.29	62.77	-0.824	nucl
ZaERF262	453	50209.21	6.74	56.83	56.45	-0.775	nucl
ZaERF40	160	17636.07	10.43	55.89	68.44	-0.634	nucl
ZaERF217	338	37628.22	8.51	45.13	66.89	-0.622	nucl
ZaERF62	178	20180.97	8.56	46.1	80.51	-0.542	chlo
ZaERF247	392	43538.5	6.09	57.96	63.29	-0.503	nucl
ZaERF120	216	23865.19	6.09	71.72	45.74	-0.904	nucl
ZaERF266	478	52130.63	7.62	54.85	56.15	-0.65	nucl
ZaERF100	203	21933.24	5.69	56.3	57.29	-0.663	nucl
ZaERF134	232	25704.56	7.8	66.27	58.06	-0.797	nucl
ZaERF103	206	22879.27	9.32	48.14	49.42	-0.896	nucl
ZaERF271	521	57440.4	6.47	48.41	63.92	-0.551	nucl
ZaERF41	161	17456.9	4	56.99	53.42	-0.477	nucl
ZaERF37	159	17237.64	4	58.79	53.46	-0.494	nucl
ZaERF71	185	20574.11	4.91	37.2	81.89	-0.314	chlo
ZaERF66	180	20079.44	7.99	53.42	69.44	-0.7	nucl
ZaERF182	297	33175.99	5.76	62.38	70.57	-0.507	nucl
ZaERF80	193	21562.17	7.8	53.2	64.77	-0.664	nucl
ZaERF85	195	21687.34	9.66	57.69	64.56	-0.613	nucl
ZaERF13	142	15550.53	9.67	48.15	60.56	-0.761	nucl
ZaERF97	200	21868.28	7.76	51.88	64.05	-0.65	nucl
ZaERF73	186	20865.18	5.04	52.44	75.65	-0.552	nucl

ZaERF111	210	23427.02	5.07	57.33	69.76	-0.582	nucl
ZaERF70	184	20707.03	5.04	52.9	75.92	-0.564	nucl
ZaERF283	958	108875.53	7.06	47.29	75.61	-0.555	nucl
ZaERF82	194	21535.11	8.56	52.37	62.42	-0.64	nucl
ZaERF11	137	15505.06	5.6	58.29	59.93	-0.776	nucl
ZaERF113	211	23544.13	5.07	55.83	68.96	-0.595	nucl
ZaERF07	127	14146.96	9.04	46.4	56.14	-0.875	nucl
ZaERF87	196	21979.67	8.59	52.49	75.26	-0.672	nucl
ZaERF135	233	26004.48	5.25	43.52	58.37	-0.808	nucl
ZaERF166	267	29881.77	5.32	42.78	55.69	-0.705	nucl
ZaERF160	257	29471.01	6.02	48.45	66.38	-0.809	nucl
ZaERF162	261	28665.73	5.76	66.61	62.8	-0.623	nucl
ZaERF19	148	16101.61	4.95	44.01	62.23	-0.535	mito
ZaERF172	276	30674.1	5.93	67.02	63.62	-0.605	nucl
ZaERF161	258	28608.97	7.02	58.47	64.3	-0.597	nucl
ZaERF122	217	24123.85	6.91	57.25	57.19	-0.622	nucl
ZaERF165	266	29225.25	5.39	69.52	61.62	-0.641	nucl
ZaERF02	111	12223.52	5.57	38.09	62.7	-0.619	nucl
ZaERF142	238	26173.55	6.74	45.81	72.27	-0.316	chlo
ZaERF08	130	14883.51	6.62	57.55	69.08	-0.832	cyto
ZaERF33	157	17926.04	9.25	40.33	60.96	-0.797	nucl
ZaERF275	565	62897.59	6.23	51.97	57.01	-0.841	nucl
ZaERF267	481	54097.1	5.75	56.53	60.64	-0.767	nucl
ZaERF57	174	19212.57	9.48	49.89	66.72	-0.717	nucl
ZaERF44	164	18513.14	9.81	54.01	72.07	-0.68	mito
ZaERF45	164	18514.13	9.71	52.84	72.07	-0.68	mito

ZaERF145	239	26133.29	6.3	34.6	62.55	-0.532	mito
ZaERF250	405	43958.42	6.34	67.9	58.17	-0.626	nucl
ZaERF258	421	47108.9	5.23	64.23	57.91	-0.822	nucl
ZaERF220	339	37638.5	4.34	60.99	64.19	-0.594	nucl
ZaERF153	249	26882.12	7.73	64.79	60.48	-0.402	nucl
ZaERF249	395	42869.98	8.63	54.22	67.72	-0.538	nucl
ZaERF214	334	36902.04	6.01	59.14	66.98	-0.547	nucl
ZaERF226	357	39445.85	5.76	62.37	66.5	-0.536	nucl
ZaERF150	244	26406.63	4.94	48.41	84.1	-0.089	chlo
ZaERF63	178	19960.55	8.63	55.55	69.66	-0.713	mito
ZaERF69	183	20590.35	8.84	53.48	72.02	-0.679	mito
ZaERF93	199	22021.84	5.25	39.02	79.95	-0.339	nucl
ZaERF91	199	21994.81	5.25	39.78	79.95	-0.326	nucl
ZaERF256	410	46227.75	7.84	46.75	54.46	-0.956	pero
ZaERF278	596	65908.03	6.24	44.03	57.8	-0.812	nucl
ZaERF55	173	19866.03	9.03	32.61	78.96	-0.617	cyto
ZaERF46	165	18384.23	9.99	50.32	89.39	-0.31	chlo
ZaERF58	174	19351.43	10.24	54.27	87.59	-0.339	chlo
ZaERF36	158	17668.4	9.9	48.88	88.42	-0.397	chlo
ZaERF279	637	71156.87	6.35	50.7	58.08	-0.767	nucl
ZaERF31	156	17964.17	9.36	46.84	54.55	-0.843	nucl
ZaERF118	215	22778.92	6.31	42.95	56	-0.645	nucl
ZaERF56	173	19397.86	9.82	62.78	75.14	-0.575	nucl
ZaERF281	681	74765.63	6.23	44.13	56.05	-0.748	nucl
ZaERF242	383	43494.33	8.11	53.15	60.18	-0.789	nucl
ZaERF243	383	43540.27	6.81	54.07	59.16	-0.789	nucl

ZaERF257	419	47032.87	8.91	47.43	59.45	-0.595	nucl
ZaERF112	211	22896.62	5.03	50.88	81.99	-0.302	chlo
ZaERF03	118	13685.05	4.83	61.46	58.81	-0.908	nucl
ZaERF191	303	33408.69	9.08	39.39	73.99	-0.436	mito
ZaERF221	345	38435.09	8.64	50.85	68.23	-0.543	nucl
ZaERF225	355	39934.83	8.64	56.24	54.23	-0.96	chlo
ZaERF04	122	14142.54	5.14	50.1	58.52	-0.97	mito
ZaERF143	239	25876.6	5.33	61.35	70.38	-0.419	nucl
ZaERF211	331	37108.69	6.04	31.1	67.79	-0.671	nucl
ZaERF222	348	38560.02	5.43	27.15	61.12	-0.61	nucl
ZaERF277	593	65878.02	6.42	45.42	55.31	-0.83	nucl
ZaERF269	494	55036.35	5.61	58.59	58.68	-0.799	nucl
ZaERF15	147	16082.18	9.71	47.94	71.9	-0.517	nucl
ZaERF196	307	34473.95	9.25	46.18	69.93	-0.605	nucl
ZaERF194	305	33158.21	7.63	56.67	65.02	-0.452	nucl
ZaERF201	318	36597.6	5.25	56.87	58.52	-0.851	nucl
ZaERF252	406	46925.56	6.26	55.71	62.88	-0.843	nucl
ZaERF251	406	46916.55	6.2	56	62.88	-0.843	nucl
ZaERF176	278	30487.22	5.44	53.82	53.06	-0.741	pero
ZaERF148	242	27458.6	6.02	50.73	65.25	-0.712	nucl
ZaERF101	205	23333.83	9.1	70.51	78.54	-0.447	extr
ZaERF106	207	22953.81	6.76	42.36	73.04	-0.505	pero
ZaERF114	212	23539.41	8.87	39.27	70.9	-0.636	nucl
ZaERF187	300	33461.97	5.75	51.62	60.43	-0.816	nucl
ZaERF90	198	22001.6	5.63	44.15	68.99	-0.588	nucl
ZaERF102	205	23349.79	8.91	70.51	77.12	-0.484	extr

ZaERF127	223	24841.9	5.4	45.24	76.1	-0.513	nucl
ZaERF184	298	33141.67	5.86	51.02	60.84	-0.799	nucl
ZaERF38	159	17831.86	5.02	64.51	69.37	-0.73	nucl
ZaERF53	172	19351.72	5.03	59.29	73.2	-0.642	nucl
ZaERF51	172	19385.74	5.03	57.42	72.62	-0.652	nucl
ZaERF27	153	17357.28	8.71	43.37	64.44	-1.003	nucl
ZaERF42	162	18317.52	8.93	44.44	63.89	-0.865	nucl
ZaERF83	194	22465.44	9.32	60.84	67.47	-0.843	chlo
ZaERF68	182	20656.28	6.34	67.3	70.27	-0.532	nucl
ZaERF95	199	21713	7.97	54.69	57.44	-0.673	nucl
ZaERF32	157	17764.7	5.84	44.12	58.47	-0.983	nucl
ZaERF12	139	15523.96	6.32	73.62	47.84	-0.969	nucl
ZaERF67	182	20677.29	6.45	68.6	71.32	-0.532	nucl
ZaERF216	336	37708.39	8.35	45.91	73.48	-0.691	nucl
ZaERF198	314	34659.79	6.7	57.13	55.73	-0.833	nucl
ZaERF263	460	51263.14	6.09	63.47	62.76	-0.683	nucl
ZaERF35	158	17551.77	9.54	42.04	69.94	-0.706	nucl
ZaERF26	153	17074.87	6.33	54.87	53.07	-0.948	nucl
ZaERF34	158	17562.36	5.98	51.99	54.49	-0.89	nucl
ZaERF138	234	26689.03	6.26	58.69	70.09	-0.611	nucl
ZaERF137	234	26582.91	6.26	57.22	70.51	-0.614	nucl
ZaERF159	257	28791.11	7.61	42.33	60	-0.805	nucl
ZaERF98	201	22556.8	5.31	46.87	49.65	-1.104	nucl
ZaERF261	436	47070.71	6.2	56.5	55.23	-0.591	nucl
ZaERF92	199	22264.35	8.91	33.84	70.7	-0.504	nucl
ZaERF229	362	40004.25	5.66	47.63	85.47	-0.395	pero

ZaERF199	315	35010.31	8.77	31.94	69.9	-0.614	nucl
ZaERF170	274	30955.32	4.83	45.09	63.03	-0.773	nucl
ZaERF156	253	27941.99	5.88	58.54	61.34	-0.686	nucl
ZaERF195	305	34044.37	5.74	68.01	50.56	-0.834	nucl
ZaERF238	379	42832.8	9.17	62.35	65.44	-0.762	nucl
ZaERF89	196	21910.07	10.04	58.11	56.33	-0.994	nucl
ZaERF207	324	36778.76	5.05	43.59	56.33	-0.995	nucl
ZaERF181	296	31507.48	8.22	42.67	52.84	-0.645	nucl
ZaERF270	501	54659.44	6.3	53.42	54.75	-0.622	nucl
ZaERF149	243	26895.15	9.35	54.39	53.13	-0.728	chlo
ZaERF124	219	24659.56	5.79	53.2	66.85	-0.682	nucl
ZaERF109	209	22693.05	9.37	48.93	59.76	-0.727	nucl
ZaERF99	202	22468.86	5.45	57.91	60.94	-0.628	nucl
ZaERF50	170	18931.09	6.85	61.56	70.65	-0.715	nucl
ZaERF152	247	28189.66	7.07	63.38	57.41	-0.853	nucl
ZaERF215	336	37581.73	5.5	58.04	67.35	-0.605	nucl
ZaERF231	362	39969.75	7.1	52.85	71.55	-0.505	extr
ZaERF130	226	25296.96	5.35	45.4	60.18	-0.779	nucl
ZaERF212	331	36272.2	5.31	53.67	67.61	-0.581	plas
ZaERF200	317	35502.21	5.27	61.82	51.36	-0.779	nucl
ZaERF01	111	12240.8	4.92	43.75	73.96	-0.352	nucl
ZaERF140	235	26053.17	5.54	72.41	62.72	-0.565	nucl
ZaERF146	240	26256.33	7.66	67.08	67.21	-0.565	nucl
ZaERF29	155	16823.86	4.21	58.45	80.06	-0.215	chlo
ZaERF30	155	16894.94	4.22	58.84	80.06	-0.235	chlo
ZaERF47	167	18615.78	4.96	40.54	65.45	-0.496	nucl

ZaERF203	319	35940.76	5.87	55.02	63.29	-0.678	nucl
ZaERF65	180	20377.84	5.54	33.96	71.06	-0.565	chlo
ZaERF132	229	25652.7	5.79	54.73	68.6	-0.628	nucl
ZaERF239	379	41589.89	4.73	64.88	53.96	-0.723	nucl
ZaERF209	326	36147.16	4.82	65.17	58.53	-0.686	nucl
ZaERF74	187	20676.81	5.45	61.05	57.49	-0.573	nucl
ZaERF49	170	18931.05	6.33	60.53	70.65	-0.712	nucl
ZaERF48	170	18932.08	6.33	60.17	70.65	-0.715	nucl
ZaERF72	186	20716.94	5.45	60.09	58.33	-0.605	nucl
ZaERF105	207	22472.83	9.02	51.43	58.94	-0.695	nucl
ZaERF236	371	41841.52	5.17	35.25	58.65	-0.728	nucl
ZaERF260	431	48377.39	8.71	46.82	61.62	-0.559	nucl
ZaERF259	431	48381.38	8.71	46.09	61.39	-0.567	nucl
ZaERF282	736	82468.01	8.08	40.22	86.93	-0.149	cyto
ZaERF155	250	28074.21	5.66	48.64	63.2	-0.701	chlo
ZaERF64	179	20145.78	7.67	50.98	65.98	-0.625	chlo
ZaERF60	177	19650.58	9.77	55.69	54.69	-0.916	nucl
ZaERF284	1020	113544.76	8.36	61.06	71.05	-0.474	nucl
ZaERF116	214	23405.45	6.9	29.03	67.1	-0.489	mito
ZaERF169	273	30658.32	9.58	36.78	68.72	-0.616	nucl
ZaERF253	407	45487.68	8.84	52.3	61.23	-0.727	nucl
ZaERF189	300	32626.81	7.88	51.39	55.33	-0.667	nucl
ZaERF09	134	15087.99	5.56	27.32	70.67	-0.412	mito
ZaERF230	362	39723.91	5.3	50.2	62.9	-0.531	nucl
ZaERF241	382	41799.82	5.05	48.69	76.86	-0.281	chlo
ZaERF28	155	16828.9	9.13	42.63	64.32	-0.539	nucl

ZaERF22	151	16726.94	9.73	45.42	64.7	-0.579	nucl
ZaERF163	263	29196.46	5.73	54.38	69.47	-0.592	nucl
ZaERF280	679	75576.74	6.15	54.7	60.57	-0.731	nucl
ZaERF213	331	36243.8	5.84	65.2	52.57	-0.752	nucl
ZaERF115	212	23088.02	8.89	59.44	61.65	-0.511	nucl
ZaERF179	290	32493.22	5.92	58.63	65.21	-0.621	nucl
ZaERF276	592	65207.19	6.27	44.98	57.04	-0.808	nucl
ZaERF223	349	38467.26	4.58	65.59	57.74	-0.775	nucl
ZaERF128	225	25212.71	9.66	55.13	62.84	-0.584	nucl
ZaERF265	468	52363.89	7.11	58.12	61.45	-0.637	nucl
ZaERF224	354	40252.87	5.14	46.97	60.62	-0.912	nucl
ZaERF186	299	33290.16	5.47	60.49	68.73	-0.637	nucl
ZaERF123	218	23805.47	8.69	60.37	63.62	-0.601	nucl
ZaERF06	125	13612.49	9.62	51.17	72.72	-0.451	nucl
ZaERF244	385	43638.95	6.87	48.35	57.04	-0.892	chlo
ZaERF208	324	36884.84	5	44.18	55.12	-1.007	nucl
ZaERF10	134	15087.99	5.56	27.32	70.67	-0.412	mito
ZaERF78	192	22110.71	10.45	64.3	38.75	-1.061	nucl
ZaERF202	318	34828.74	5.1	50.26	53.43	-0.751	pero

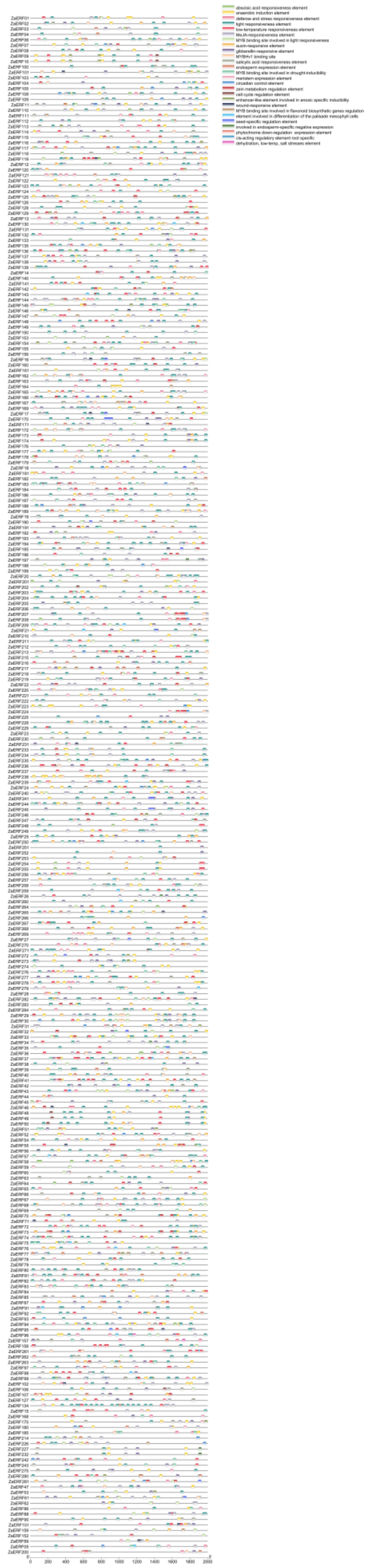

Figure S7. Cis-acting regulatory elements in the promoters of *ZaAP2/ERF* genes.

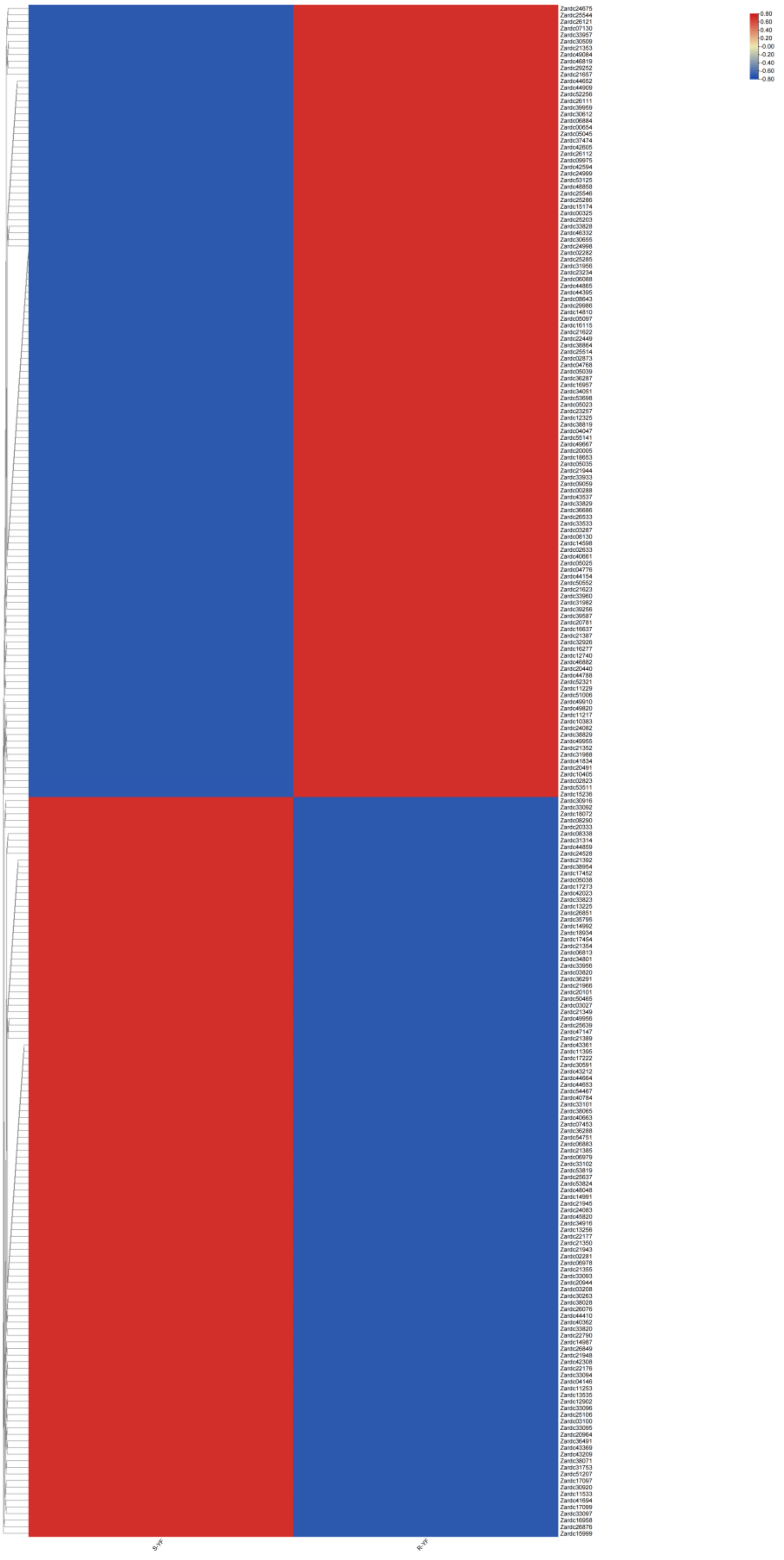

Figure S8. Heatmap of differential expression of 232 *ZaAP2/ERF* genes in leaves of the resistant line R-YF and the susceptible line S-YF. Color blocks represent gene expression levels as log_2FC; red indicates up-regulation in R-YF, and blue indicates higher expression in S-YF.
